# Major Clades of Australasian Rutoideae (Rutaceae) Based on *rbc*L and *atp*B Sequences

**DOI:** 10.1371/journal.pone.0072493

**Published:** 2013-08-13

**Authors:** Michael J. Bayly, Gareth D. Holmes, Paul I. Forster, David J. Cantrill, Pauline Y. Ladiges

**Affiliations:** 1 School of Botany, The University of Melbourne, Parkville, Victoria, Australia; 2 Queensland Herbarium, Department of Science, Information Technology, Innovation and the Arts, Brisbane Botanic Gardens, Toowong, Queensland, Australia; 3 National Herbarium of Victoria, Royal Botanic Gardens Melbourne, South Yarra, Victoria, Australia; Montreal Botanical Garden, Canada

## Abstract

**Background:**

Rutaceae subfamily Rutoideae (46 genera, c. 660 species) is diverse in both rainforests and sclerophyll vegetation of Australasia. Australia and New Caledonia are centres of endemism with a number of genera and species distributed disjunctly between the two regions. Our aim was to generate a high-level molecular phylogeny for the Australasian Rutoideae and identify major clades as a framework for assessing morphological and biogeographic patterns and taxonomy.

**Methodology/Principal Findings:**

Phylogenetic analyses were based on chloroplast genes, *rbc*L and *atp*B, for 108 samples (78 new here), including 38 of 46 Australasian genera. Results were integrated with those from other molecular studies to produce a supertree for Rutaceae worldwide, including 115 of 154 genera. Australasian clades are poorly matched with existing tribal classifications, and genera *Philotheca* and *Boronia* are not monophyletic. Major sclerophyll lineages in Australia belong to two separate clades, each with an early divergence between rainforest and sclerophyll taxa. Dehiscent fruits with seeds ejected at maturity (often associated with myrmecochory) are inferred as ancestral; derived states include woody capsules with winged seeds, samaras, fleshy drupes, and retention and display of seeds in dehisced fruits (the last two states adaptations to bird dispersal, with multiple origins among rainforest genera). Patterns of relationship and levels of sequence divergence in some taxa, mostly species, with bird-dispersed (*Acronychia*, *Sarcomelicope*, *Halfordia* and *Melicope*) or winged (*Flindersia*) seeds are consistent with recent long-distance dispersal between Australia and New Caledonia. Other deeper Australian/New Caledonian divergences, some involving ant-dispersed taxa (e.g., *Neoschmidia*), suggest older vicariance.

**Conclusions/Significance:**

This comprehensive molecular phylogeny of the Australasian Rutoideae gives a broad overview of the group’s evolutionary and biogeographic history. Deficiencies of infrafamilial classifications of Rutoideae have long been recognised, and our results provide a basis for taxonomic revision and a necessary framework for more focused studies of genera and species.

## Introduction

Rutaceae are a widespread (sub-cosmopolitan) family of flowering plants in the order Sapindales [1]. The family includes the commercially important genus *Citrus*, common rue (*Ruta graveolens*), curry leaf tree (*Murraya koenigii*), some regionally-used timber trees (e.g., species of *Amyris*, *Chloroxylon*, *Flindersia*, *Zanthoxylum*), the sources of Angostura bitter (*Angostura trifoliata*) and Sichuan pepper (*Zanthoxylum* spp.), as well as some widely-used ornamental plants including *Diosma*, *Correa*, *Choisya*, *Murraya* and *Boronia*. Worldwide there are c. 2,100 species and 154 genera [2], and the family has major centres of endemism in Australia and southern Africa, especially in sclerophyllous vegetation.

**Figure 1:**
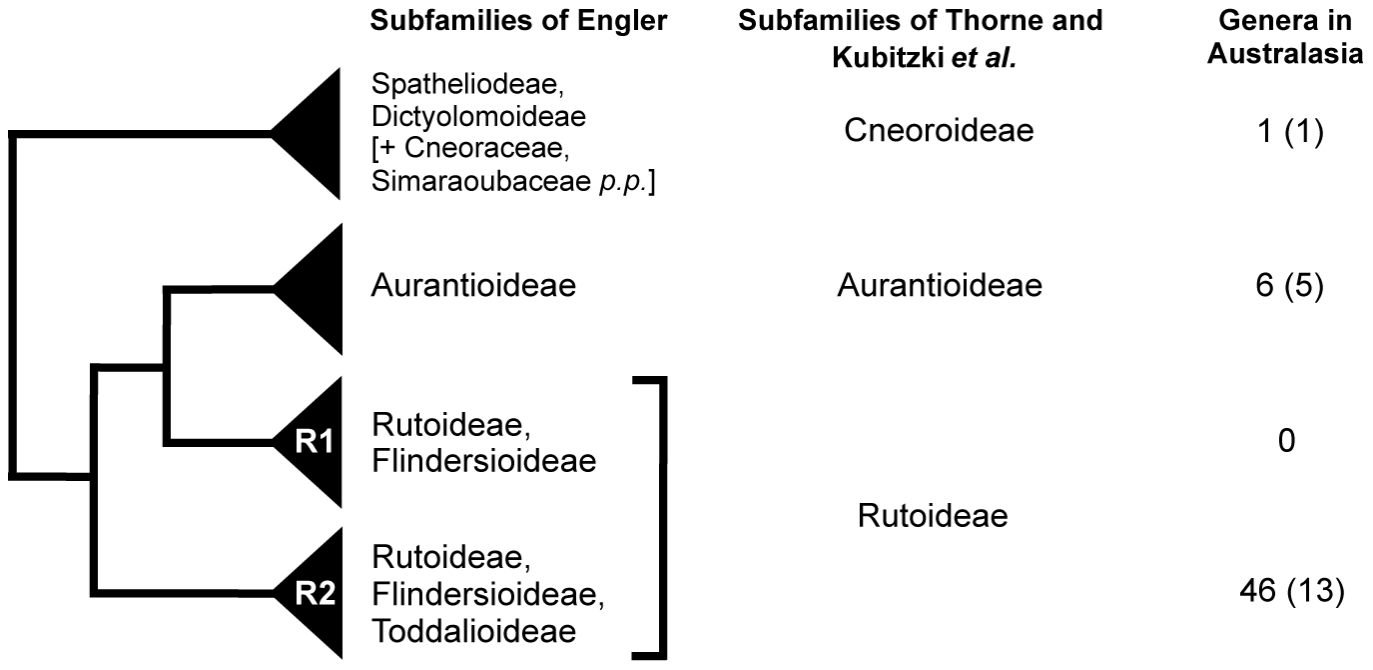
Summary of the major clades of Rutaceae, based on published molecular phylogenies[16], [19]. Shown for comparison are the subfamilial classifications of Engler [3], [4], Thorne [20] and Kubitzki *et al.* [2]. The number of Australasian genera in each group is indicated (values in brackets represent the total number of those genera included in family-wide molecular phylogenies [16], [19]). Subfamily Rutoideae, as currently defined, is paraphyletic; its type genus, *Ruta*, occurs in clade R1. If clade R2 was considered worthy of recognition as a separate subfamily it would need a different name: the oldest of the names available, each with equal priority under the International Code of Botanical Nomenclature [65], are Zanthoxyloideae, Amyridoideae and Diosmoideae.

A landmark work on the classification of Rutaceae was that of Engler [3], [4] wherein the family was divided into seven subfamilies, which were further divided into tribes and subtribes. Engler’s classification put substantial emphasis on characters of ovaries and fruit, in particular the degree of fusion of the carpels, and whether fruits were dehiscent or indehiscent. Morphological [5], [6], [7], [8], [9], [10], phytochemical [11], [12], [13], [14], cytological [15] and molecular phylogenetic studies [16], [17], [18], [19] have highlighted problems with Engler’s classification and, currently, there are no subfamilial, tribal or subtribal classifications for the family that fully reconcile with molecular phylogenies. Here, for the sake of convenience, we follow recent subfamilial classifications [2], [20], with three subfamilies recognized (Cneoroideae, Aurantioideae, Rutoideae), even though one of these (Rutoideae) is demonstrably paraphyletic (Fig. 1).

**Figure 2:**
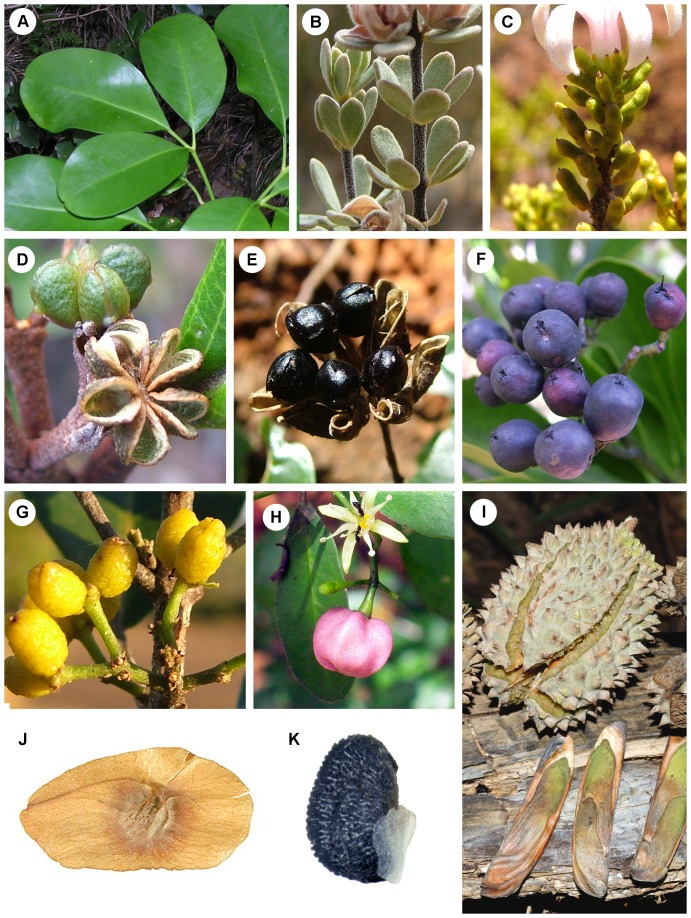
Examples of variation in Australasian Rutoideae in leaves, fruits and seeds. **A**, opposite, compound leaves of rainforest tree *Melicope polybotrya*. **B**, opposite, compound leaves of xeromorphic shrub *Boronia ternata*. **C**, alternate, simple and terete leaves of *Philotheca tubiflora*. **D**, dehiscent fruit (follicles) of *Myrtopsis*, from which the seeds and endocarp are forcibly ejected at maturity. **E**, fruit and seeds of *Melicope glaberrima*. The seeds are adapted for bird dispersal. At maturity they remain attached to the follicles (“displayed” in the canopy). They have a shiny outer layer (pellicle), below which there is a spongy layer (sarcotesta) surrounding a strong inner layer of the seed coat (sclerotesta) capable of withstanding endozoochory. **F**, syncarpous drupes of *Halfordia kendack* (i.e., each drupe is the product of a single flower). **G**, apocarpous drupes of *Comptonella microcarpa* (i.e., each drupe is the product of a single carpel, with multiple drupes per flower). **H**, syncarpous drupe of *Acronychia laevis*. **I**, woody capsule of *Flindersia australis,* with winged seeds. **J**, winged samara of *Pentaceras australis*. **K**, seed of *Philotheca difformis*. An adaxial portion of endocarp remains attached to dehisced seeds and is assumed to function as an elaiosome for ant dispersal. [Credits: A, P. Ladiges; H, Peter Woodward; I, N. Turland; J, USDA-ARS, U.S. National Arboretum, U.S. National Seed Herbarium, Washington, DC, prepared by Robert J. Gibbons for [126]; B, C, D, E, F, G, K, M. Bayly.]

Subfamily Rutoideae is, by far (Fig. 1), the largest group of Rutaceae in Australasia, a region which here includes Australia, New Guinea, New Zealand and New Caledonia. In total the Australasian members of Rutoideae include 46 genera and c. 660 species (Table 1). They are morphologically and ecologically diverse, e.g., ranging from rainforest trees and woody lianes to small-leaved shrubs of subalpine or semi-arid areas. They also display a diversity of fruit and seed morphology (Fig. 2), including fleshy, bird-dispersed drupes, indehiscent samaroid fruits, and dehiscent fruits that release seeds variously adapted to dispersal by ants, birds, or wind. The majority of genera occurring in Australasia are endemic to the region, although some, mostly rainforest genera, are more widespread in Malesia, the Pacific, or beyond (Table 1). Within Australasia the group is biogeographically interesting, e.g., with many genera, and some species, shared between Australia and New Caledonia. The family, based on fossils [21], [22] and some molecular dating studies [23], [24], has been suggested to date from the Late Cretaceous, and is thus a candidate for having a vicariant gondwanan history.

**Table 1:** Genera of Australasian Rutaceae: geographic distributions and species numbers.

Genus	Distribution	No. of species (no. of species sampled here)
*Acradenia* [53]	Australia	2 (2)
*Acronychia* [9], [27], [33], [127], [128], [129]	India, S Asia, Malesia, Australia, New Caledonia, Pacific Ids	48 (4)
*Asterolasia* [130]	Australia	18 (1)
*Boronella* [31]	New Caledonia	c. 6 (2)
*Boronia* [2]	Australia	c. 148 (2)
*Bosistoa* [7], [130]	Australia	4 (2)
*Bouchardatia* [7]	Australia	1 (1)
*Brombya* [7], [130]	Australia	2 (2)
*Chorilaena* [2]	Australia	1 (1)
*Coatesia* [7], [130]	Australia	1 (1)
*Comptonella* [26], [63]	New Caledonia	8 (2)
*Correa* [26], [131]	Australia	11 (2)
*Crossosperma* [10]	New Caledonia	2 (1)
*Crowea* [46], [132], [133]	Australia	3 (3)
*Dinosperma* [10]	Australia	4 (2)
*Diplolaena* [134]	Australia	15 (2)
*Drummondita* [135], [136], [137]	Australia	9 (1)
*Dutailliopsis* [10]	New Caledonia	1 (–)
*Dutaillyea* [61]	New Caledonia	2 (1)
*Eriostemon* [37], [138]	Australia	2 (1)
*Euodia* [22]	New Guinea, Australia, New Caledonia, east to Samoa, Tonga, Niue	7 (2)
*Flindersia* [139], [140], [141]	Moluccas, New Guinea, New Caledonia (1 sp.), Australia	17 (5)
*Geijera* [7]	New Guinea, Australia, New Caledonia	c. 6 (3)
*Geleznowia* [41], [42]	Australia	1 (1)
*Halfordia* [7], [43]	New Caledonia, New Guinea, New Britain, Australia, Vanuatu	1 (1)
*Leionema* [2], [142], [143]	Australia, New Zealand (1 sp.)	c. 26 (1)
*Lunasia* [7]	Philippines to Java, New Guinea, Australia	1 (1)
*Medicosma* [7], [116]	Australia, New Guinea, New Caledonia	25 (2)
*Melicope* [22]	Madagascar, India, S China, Malesia, Australia, New Zealand, Norfolk Id, Pacific Ids	233 (6)
*Microcybe* [2]	Australia	c. 4 (–)
*Muiriantha* [2]	Australia	1 (–)
*Myrtopsis* [7]	New Caledonia	c. 9 (2)
*Nematolepis* [143]	Australia	7 (1)
*Neobyrnesia* [34]	Australia	1 (1)
*Neoschmidia* [32]	New Caledonia	2 (1)
*Pentaceras* [2]	Australia	1 (1)
*Perryodendron* [10]	Moluccas, New Guinea and New Britain	1 (–)
*Phebalium* [2], [46], [143]	Australia	c. 28 (2)
*Philotheca* [35], [37], [144], [145], [146], [147]	Australia	53 (5)
*Picrella* [64]	New Caledonia	3 (3)
*Pitaviaster* [10]	Australia	1 (1)
*Rhadinothamnus* [2]	Australia	3 (–)
*Sarcomelicope* [62], [148]	Australia to Fiji, including New Caledonia	9 (2)
*Tetractomia* [115]	Malesia, including New Guinea	6 (–)
*Zanthoxylum* [7]	Pantropical	c. 200 (5)
*Zieria* [149], [150], [151]	Australia, New Caledonia (1 sp.)	60 (3)
	**Total**	c. 994 (82)

Molecular phylogenetic studies [16], [18], [19] have provided new insights into relationships among the Australasian Rutoideae, but they have not been extensive. The *Phebalium* group [25], *Correa* [26], *Acronychia* [27] and *Flindersia* [18] have been the subjects of focused investigations. Six Australasian rainforest genera were included in studies of various Rutoideae and Toddalioideae [28], [29], and four Australasian species were included in a study of *Melicope* in the Pacific [30]. Sampling in broader studies has otherwise been limited, including only up to c. 13 of the 46 genera [19], and no studies have sampled taxa from New Caledonia.

Additional phylogenetic data for Australasian Rutoideae is essential for understanding patterns of morphological evolution, habitat shifts between rainforests and more xeric environments, biogeographic history within Australia and across Australasia, and for revising the classification of the group at a range of levels, from genus to tribe. Recent decades have seen substantial rearrangements of generic boundaries for New Caledonian [10], [31], [32] and Australian rainforest [10], [33] and sclerophyllous groups [34], [35], [36], [37] that need to be tested with molecular data. Tribal limits in Australasian Rutoideae have been a subject of debate [5], [31], [32], and molecular phylogenies [19] have highlighted clear problems with all proposed schemes, requiring further studies with additional taxon sampling.

The present study investigates phylogenetic relationships of the Australasian Rutoideae using sequences from the chloroplast genes *rbc*L and *atp*B. This combination of genes is mostly informative at taxonomic levels above the rank of genus. It was used in an earlier, family-wide study [16], which we have expanded here with sequences from an additional 78 samples, representing 73 species and 38 of 46 Australasian genera. Our aim was to identify major clades in the group in order to provide a framework for: a) assessing broad-scale morphological and biogeographic patterns; b) revising classifications, especially at ranks of subfamily, tribe and subtribe; c) identifying monophyletic groups that can be the focus of future lower-level phylogenetic, taxonomic and biogeographic studies using more rapidly mutating DNA markers.

## Results and Discussion

### Phylogenetic analyses: Major Clades of Australasian Rutoideae

**Figure 3:**
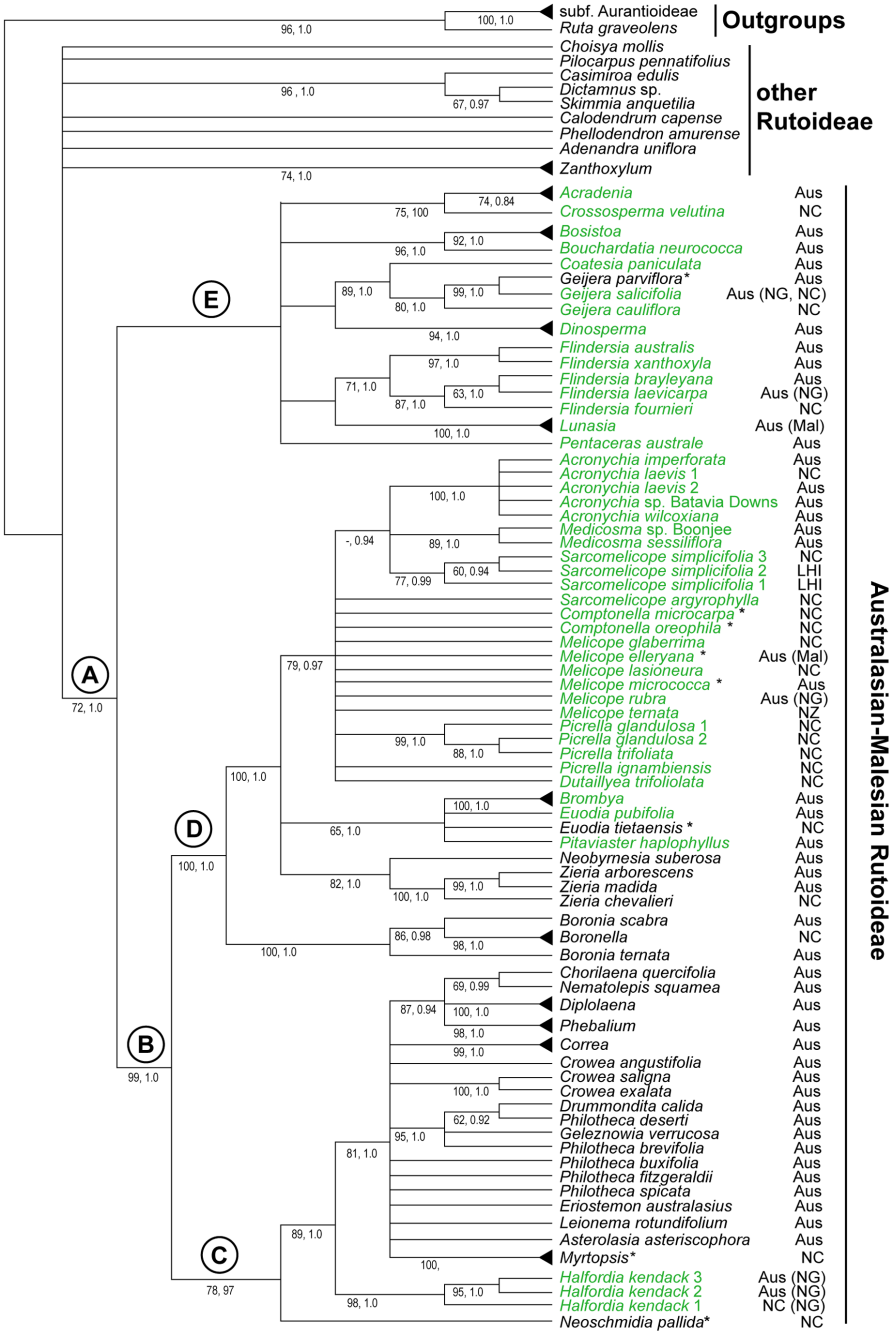
Strict consensus of trees produced by MP analysis of combined *rbc*L and *atp*B sequences. Details have been simplified for some clades (indicated by terminal triangles). The most parsimonious trees were 1277 steps long (958 steps excluding autapomorphies), with CI  =  0.59 and RI  =  0.81. Numbers below branches are MP bootstrap values, followed by posterior probability values from BI analysis of the same dataset. Distribution information is shown to the right of taxon names; areas shown in brackets are not represented by samples in this study. Taxa in green occur chiefly in rainforests. Asterisks denote taxa that occur in a range of habitats, sometimes including rainforests. Abbreviations: Aus, Australia; LHI, Lord Howe Island; Mal, Malesian region; NC, New Caledonia; NG, Indonesian Papua and Papua New Guinea.

**Figure 4:**
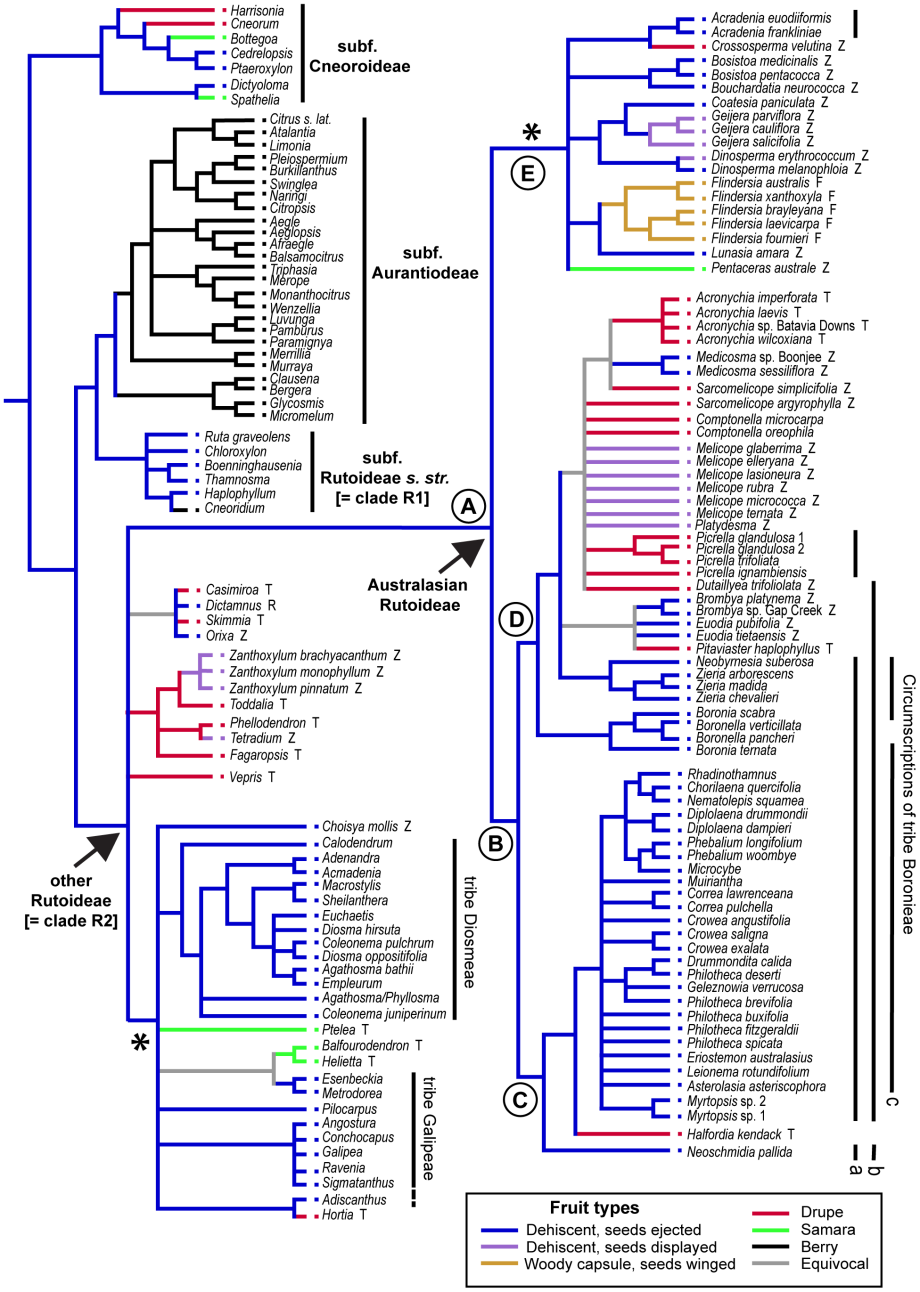
Supertree summarising relationships in Rutaceae, combining results of the present study with those of other molecular phylogenies (see Methods for details). The tree includes 115 of the 154 genera currently recognised [**2**]. The distribution of fruit types is optimised on the tree (colour coding of branches). Also shown are the limits of subfamilies and some tribes, including circumscriptions of the tribe Boronieae by: a, Engler [3], [4]; b, Armstrong [5]; c, Hartley [31], [32]. Clades R1 and R2 match those of Fig. 1; clades A-E match those of Fig. 2. Letter codes following taxon names in clade R2 indicate other tribal placements in the classification of Engler [3], [4] (Z, Zanthoxyleae; R, Ruteae; T, Toddalieae; F, Flinderseae). Asterisks denote clades with <50% bootstrap support in analyses of the primary data.

**Figure 5:**
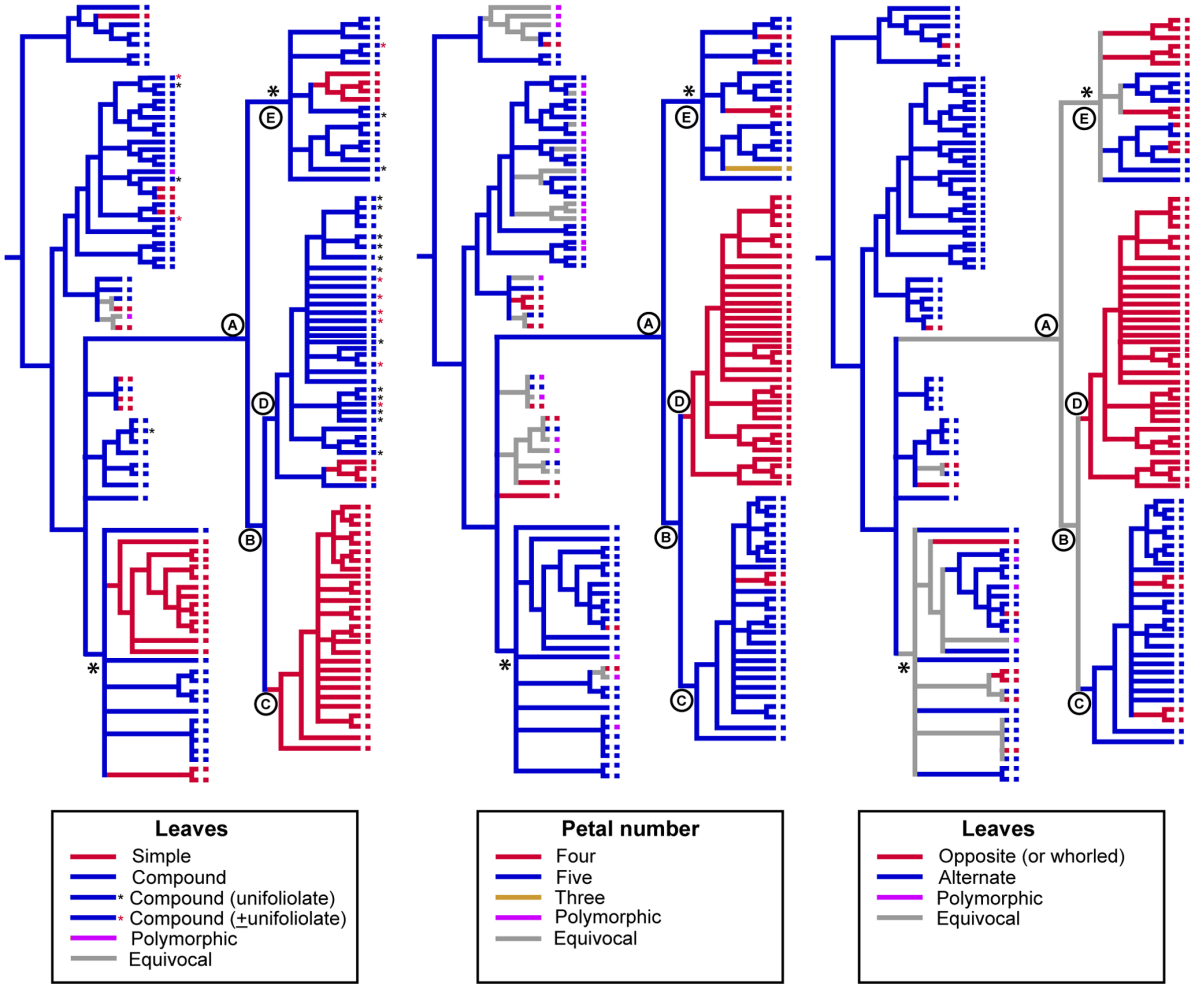
Leaf and flower characters mapped on to supertree for Rutaceae. Tree topology and clade labels match those of Fig. 3.

The combined *rbc*L and *atp*B dataset included 619 variable characters, of which 351 were parsimony informative (180 from the *rbc*L dataset, 171 from *atp*B). Phylogenetic analyses based on maximum parsimony (MP) and Bayesian inference (BI) produced very similar results, with no incongruence between nodes that were well-supported in either analysis. The MP strict consensus tree (Fig. 3) has BI support values mapped on to it and an MP phylogram with branch lengths is shown in Fig. S1. Results from the present study were combined with those from previous studies, in the form of a supertree (Fig. 4), to provide an overview of phylogenetic relationships across the whole family as inferred from molecular phylogenies (only taxa in published molecular phylogenies are included in this supertree). The distributions of some key morphological characters are mapped onto this tree (Figs 4, 5).

With the exception of the pantropical genus *Zanthoxylum*, the Australasian Rutoideae were placed together in the analyses (clade A; Fig. 3) with moderate to strong support from MP bootstrap (BS; 72%) and BI posterior probability (PP; 1.0). The Australasian group was further divided into three main clades in the MP strict consensus tree. Two of these, clades C and D (Fig. 3), are each moderately to strongly supported (BS/PP of 78/0.97 and 100/1.0, respectively) and are strongly supported as sister clades (clade B; BS/PP, 99/1.0). The third main group, clade E, although present in the strict consensus from the MP analysis (Fig. 3), has less than 50% BS and is supported by just a single, non-synonymous (1^st^ codon position) but homoplasious substitution in *rbc*L. This clade was also absent in the BI majority rule consensus tree, although some members of the clade grouped in a trichotomy (*Lunasia*, *Flindersia* and *Coatesia* + *Geijera*), but with low support for the node, PP 0.71.

The major clades recovered here are consistent with results of previous molecular phylogenetic analyses of Rutaceae [16], [19]. For example, Groppo *et al.* [19] recovered a *Boronia*/*Melicope*/*Sarcomelicope*/*Medicosma*/*Acronychia* clade, representing our clade D; the additional taxa added here being *Euodia*, *Pitaviaster*, *Brombya*, *Medicosma*, *Comptonella*, *Picrella*, *Dutaillyea*, *Zieria*, *Neobyrnesia* and *Boronella*. They [19] also recovered a sister clade consisting of *Halfordia* + *Correa* + *Diplolaena* + *Chorilaena* + *Nematolepis*, representing our clade C, to which we have added another 10 genera. Sister to these groups in previous analyses [16], [19] was the group *Flindersia* + *Lunasia*, representing clade E here.

Clades C–E each represent diverse assemblages of taxa in terms of their vegetative and reproductive morphology, habitat and ecology, but there is broad correlation, at least for the well-supported clades C and D, with some morphological traits. Clade C is comprised mostly of shrubby taxa of open forests or shrublands (the exception being *Halfordia*) that have 5-merous flowers (4-merous in *Correa*) and simple, alternate leaves (opposite in *Correa* and *Myrtopsis*). Members of clade D are mostly rainforest plants (except the early diverging lineages *Boronia*, *Boronella*, *Zieria* and *Neobyrnesia*) that have 4-merous flowers and compound/unifoliate, opposite/whorled leaves (simple in *Neobyrnesia*, *Boronella* and some species of *Boronia*, Fig. 2B). The poorly-supported clade E includes mostly rainforest plants (except *Geijera parviflora* and one species of *Flindersia*, not sampled) with compound leaves (except *Coatesia* + *Geijera*); flower merosity and leaf arrangement are variable.

Simple leaves appear to be a synapomorphy of clade C, and 4-merous flowers a synapomorphy of clade D (Fig. 5). However, these character optimisations should be treated with caution. This is partly because taxon sampling (e.g., incomplete species sampling in the Australasian group) obscures some morphological variation, and because further resolution of polytomies, or collapse of poorly supported nodes, could affect interpretation of some characters.

### Placement of unsampled genera

Sampling for this study has focused on the Rutoideae of Australia and New Caledonia. In terms of these areas, sampling at generic level in the supertree is almost complete; the only genus now unsampled in molecular phylogenetic studies, the monotypic *Dutaillopsis* from New Caledonia, is allied to *Dutaillyea* on morphological grounds [10] and, on the basis of its 4-merous flowers and opposite, compound leaves, it would be reasonably placed with *Dutaillyea* in clade D.

A geographic sampling focus, rather than a phylogenetic or taxonomic one, runs the risk, through omission of taxa, of providing a picture of relationships or inferred evolutionary or biogeographic patterns that is potentially misleading. In the case of Rutoideae it seems likely that there are only a small number of unsampled genera from other areas that might nest within the Australasian group (clade A). The most likely are the monotypic South American genus *Pitavia* (see below under “Relationships of Clade E”) and genera from the *Euodia* alliance (*sensu* Kubitzki *et al.* [2]), namely *Ivodea* (c. 10 species from Madagascar and the Comoros Archipelago [2], [38]) and the Malesian genera *Maclurodendron* (6 species), *Perryodendron* (1 species) and *Tetractomia* (9 species). On morphological grounds, *Maclurodendron* is suggested to be related to *Melicope* and *Sarcomelicope* [7], whereas *Perryodendron* and *Tetractomia* are considered closely related to *Euodia* [7], [10]. *Ivodea*, *Maclurodendron*, *Perryodendron* and *Tetractomia* have 4-merous flowers and opposite, compound (unifoliolate leaves), which would be consistent with placement in clade D.

### Classification of Tribes

As long acknowledged [5], [6], [9], [11], [13], classification of Rutaceae at the ranks of tribe and subtribe is clearly in need of revision, and this is further supported by the results here (Fig. 4). Within Rutoideae (clade R2), tribes Zanthoxyleae, Toddalieae and Boronieae *sensu* Engler [3], [4] are all polyphyletic, in line with previous molecular phylogenies [16], [18], [19].

Of particular interest in Australia is the tribe Boronieae. Various classifications have been proposed for this group [3], [4], [5], [31], [32], but the name Boronieae has generally been used to refer to the species-rich group of Australian taxa that are mostly sclerophyllous shrubs that occur outside rainforests, or occasionally on their ecotonal margins with sclerophyll communities. The most recent circumscription [31], [32] used features of the cotyledons to define the group, with Boronieae having linear cotyledons that are about the same width as the hypocotyl, compared with other groups that have cotyledons considerably wider than the hypocotyl. Analyses based on *trn*L-F and *rps*16 sequences [19] showed *Boronia* (the type of the tribe) separated from other members of the group. That result is further supported by the present study, wherein *Boronia* (as well as *Boronella*, *Zieria* and *Neobyrnesia*, all of which have been placed in Boronieae at some stage) is placed in clade D, separated from other Boronieae in clade C (Fig. 4). This suggests that cotyledon features are homoplasious, with linear cotyledons being derived at least twice. It is tempting to speculate that there might be a selective advantage of narrow cotyledons that explains the correlation of this feature with drier habitats and low nutrient soils, although some counter arguments to this view have already been presented [31].

A revised tribal (and subtribal) classification of Australasian Rutoideae based on monophyletic and morphologically-diagnosable groups would be worthwhile. Such a classification should be constructed with adequate consideration of the relationships and classification across the family as a whole, so that taxonomic ranks are used in a consistent way. With the exception of the neotropical taxa of subtribe Galipeinae [2], [39], [40] (6 of 26 genera sampled) and the *Polyaster* alliance [2] (0 of 5 genera sampled), most major lineages of Rutoideae are now well-sampled in molecular phylogenetic studies. The supertree here includes 115 of 154 genera, and a proportion of unsampled genera could readily be assigned to major groups based on morphology. However, there are still some parts of the phylogeny that are either poorly resolved or poorly supported. For instance, the relationships among Australasian members of clade E are poorly supported, as are those between members of tribes Galipeeae and Diosmeae (Fig. 4). Such uncertainties limit the confidence with which we could devise a new tribal or subtribal classification based on monophyletic groups, and it is premature to attempt a formal classification (using ranks of the International Code of Botanical Nomenclature) at this stage.

If tribal groups were recognised in the Australasian Rutoideae (and it is debatable at what level in the phylogeny the use of tribal names might be appropriate), the strict application of existing tribal names could have some undesirable consequences. The two tribal names that are typified by members of Australasian Rutoideae (clade A) are Boronieae Bartl. (dating from 1830; type *Boronia* Sm.) and Flindersieae Burnett (dating from 1835; type *Flindersia* R.Br.). Both of these names have a long history of use, and any new circumscriptions could differ substantially from traditional usage. For instance, whether a new classification used broad (e.g., placing all of clade A in one tribe, Boronieae) or narrow (limiting Boronieae to clade D or part thereof) tribal limits, it would necessarily use the name Boronieae in a manner mismatched with historical literature. Such implications should at least be considered when devising a new classification for the group.

### Genus-level Taxonomy: including *Philotheca*, *Boronia* and *Melicope*

Of the Australasian genera included in the analysis, nine are currently recognized as monotypic (*Bouchardatia*, *Chorilaena*, *Coatesia*, *Geleznowia*, *Lunasia*, *Neobyrnesia*, *Pentaceras*, *Pitaviaster, Halfordia*), although two of them include geographic variants that could potentially warrant taxonomic recognition [41], [42], [43]. Among these, *Geleznowia* (along with the genus *Drummondita*) is shown, with strong support (95%, 1.0), as nested within *Philotheca*, i.e., members of sect. *Philotheca* (*P. deserti* and *P. brevifolia*) are shown as more closely related to *Geleznowia* and *Drummondita* than they are to other sections of the genus. The phylogeny provides no evidence that any of the eight other monotypic genera might be nested within broader generic groups, although there is strong support, based on current sampling, for the sister relationships of some of them, including, in line with expectations based on morphology [7], [34], [44], [45]: *Neobyrnesia* to *Zieria*, *Bouchardatia* to *Bosistoa*, *Coatesia* to *Geijera*.

Most genera for which two or more species were included in the sampling were resolved as monophyletic. These genera are *Acradenia* (2 of 2 species), *Acronychia* (4 of 48 species), *Medicosma* (2 of 25 species), *Brombya* (2 of 2 species), *Zieria* (3 of 60 species), *Phebalium* (2 of 28 species), *Diplolaena* (2 of 15 species), *Correa* (2 of 11 species), *Myrtopsis* (2 of c. 9 species), *Bosistoa* (2 of 4 species), *Geijera* (3 of c. 6 species), *Dinosperma* (2 of 4 species), *Flindersia* (5 of 17 species), and *Zanthoxylum* (5 of c. 200 species). The analysis was equivocal regarding the monophyly of *Comptonella*, *Melicope*, *Picrella*, *Euodia*, and *Crowea*. In each of these cases members were placed in unresolved polytomies with those of other genera, although clearly monophyletic subgroups were resolved for some taxa, e.g., *Crowea* and *Picrella*. Taxa of *Sarcomelicope* were separated in the MP strict consensus, but the intervening node had <50% BS and <0.95 PP, so this split is not strongly supported.

Two Australian genera were identified as non-monophyletic: *Philotheca* (mentioned above) and *Boronia*. Sampling for *Boronia* was sparse (2 of c. 148 species), but there is good support (BS 86%; PP 0.98) that the New Caledonian genus *Boronella* (2 of c. 6 species sampled) is nested within it. The limits of *Philotheca* and allied genera have had substantial revisions in recent decades [35], [36], [37], [46], and even the latest of these revisions [37], based on morphological comparisons, hinted that *Philotheca* might not be monophyletic and in particular that the monotypic genus *Geleznowia* could be nested in it. Similarly, there has been debate over the delimitation of *Boronia* and *Boronella* [31], [47], and the results here are consistent with those of a previous morphological phylogenetic analysis that suggest *Boronella* is nested within *Boronia* [47]. Further phylogenetic analyses of *Philotheca*, *Boronia* and allied genera, using greater taxon sampling and more rapidly mutating DNA markers would be worthwhile to resolve the relationships and appropriate taxonomy for these groups.

Relationships in clade D (Fig. 3), in particular among the members of *Melicope*, are not well-resolved. Previous analyses based on more rapidly mutating ITS and *trn*L-F sequences [28], [29] sampled species of *Acronychia* and *Euodia*, along with seven species of *Melicope*, of which *M. elleryana*, *M. rubra* and *M. ternata* were duplicated here. Those markers provided better resolution of relationships, and placed species of *Melicope* in two clades: five species formed a monophyletic group, but *M. vitiflora* was placed in a distinct clade with *Euodia*. Thus the generic limits in *Melicope* warrant further investigation. The previous studies [28], [29] indicate that ITS and *trn*L-F sequences could be suitable for this purpose, and the present study shows that inclusion of additional genera, in particular *Medicosma*, *Sarcomelicope*, *Comptonella*, *Picrella* and *Dutaillyea*, is necessary for proper consideration of relationships in this group.

### Divergence Between Lineages of Rainforest and Sclerophyll Vegetation

In Australia there is a fairly clear division between Rutoideae that occur in rainforests and those of other vegetation types. Australian rainforests, including littoral rainforest and vine thickets [48], [49], are closed forests with a canopy cover > 70%, usually on comparatively fertile soils at sites of suitable rainfall. Early in the Cenozoic rainforests were widespread in Australia [50], [51], but they contracted dramatically in area, especially in the Neogene, and are now restricted mostly to the eastern margin of the continent and in suitable areas of monsoonal northern Australia [48]. Rainforest Rutoideae occur chiefly in the tropical, subtropical and warm temperate forests of eastern Australia, *sensu* [48], although one genus (*Acradenia*) has an outlying, disjunct species, *A*. *frankliniae*, that occurs further south in the cool temperate rainforests of Tasmania. Given both the vegetation history of Australia [48], [49], and the pattern of relationships inferred here (Fig. 3), it seems plausible [52] that the Australian Rutoideae were ancestrally rainforest plants, and that some lineages adapted to other habitats that became more widespread in the Cenozoic.

Most Australian Rutoideae occurring outside rainforests (those of more xerophytic or sclerophyllous vegetation) belong to three groups, placed in two of the major clades recovered here (Fig. 3), namely: *Neobyrnesia* + *Zieria* (clade D), *Boronia* + *Boronella* (clade D), the large clade sister to *Halfordia* (clade C). The lineage in clade C is by far the largest (representing c. 15 genera and 182 species in Australia and one from New Zealand), followed by *Boronia* (c. 148 Australian species, with 4 species of *Boronella* in New Caledonia) and *Zieria* (59 Australian species, plus one in New Caledonia) and *Neobyrnesia* (1 species, endemic to the Northern Territory, Australia). Outside of clades C and D, non-rainforest taxa include some species of *Geijera* (*G. parviflora* and the unsampled *G. linearifolia*) and *Flindersia* (*F. maculosa* and *F. dissosperma*, both unsampled here), which are placed in clade E.

Relationships among Australian Rutoideae suggest that only a limited number of habitat shifts between rainforests and other vegetation types can be inferred, at least as revealed by extant lineages. Independent habitat shifts can be inferred in *Flindersia*, *Geijera* (both clade E) and in each of the larger groups of clades C and D. In clades C and D, even though the relationships of *Zieria* + *Neobyrnesia* are somewhat unresolved, it is evident there are deep divergences between rainforest and sclerophyll lineages, suggesting early differentiation of these groups without further shifts, at least in Australia, between the two habitat categories. The situation for New Caledonia is more complex with a number of genera and some species variably present in both rainforest and in more open maquis vegetation on ultramafic substrates (e.g., *Euodia*, *Comptonella*, *Neoschmidia* and *Myrtopsis*; Fig. 3), and better understanding of habitat divergences among New Caledonian lineages will require greater levels of phylogenetic resolution and taxon sampling than provided here.

### Relationships of Clade E: including *Acradenia* and *Crossosperma*

Within clade E (Fig. 3) there is good support for the monophyly of genera and for a close relationship of *Coatesia* + *Geijera*. However, the relationships among most genera are not well-resolved or supported in either the MP analysis or the BI analysis, which groups only a subset of taxa (*Lunasia*, *Flindersia*, *Coatesia* + *Geijera*) with poor support (PP of 0.71). As discussed above, taxa of clade E are mostly rainforest plants but are diverse in terms of their leaf type, leaf arrangement, fruit/seed morphology and petal number, among other floral and vegetative characters. One feature that is shared by a number of these genera (*Bosistoa*, *Bouchardatia*, *Acradenia*, *Dinosperma*, *Lunasia*, *Crossosperma*, *Flindersia*) is the lack of a hard, inner layer (sclerotesta) in the seed coat. The data provided by Hartley [7] suggest that lack of a sclerotesta (versus presence in all other Australian Rutoideae) is apomorphic. Thus, on balance, it seems likely that at least some of the genera placed in clade E are related. Understanding the pattern of these relationships will require further evidence (molecular and/or morphological) and will have implications for infrafamilial taxonomy and inferences of character evolution (e.g., leaf arrangement, Fig. 5).

One relationship within clade E that is moderately to well-supported (75, 1.0) is that between the Australian genus *Acradenia* (2 of 2 species sampled) and the New Caledonian genus *Crossosperma* (1 of 2 species sampled; the unsampled species, *C. cauliflora*, is known only from the type locality and has not been relocated since it was last collected in 1976, despite comprehensive surveys of the area by J. Munzinger and colleagues). Both genera have been considered to have isolated positions in the family, without obviously close relatives [10], [53], and a relationship between them has not been suggested. They both have leaves that are opposite and compound, and seeds that lack a sclerotesta, but they differ in many features of flowers and fruits: e.g., *Acradenia* [53] has mostly 5-merous flowers, ovaries that are only basally connate and each capped with a prominent gland, 2 ovules per ovary, stigmas that are scarcely differentiated from the style, and follicular fruits that release wingless seeds, whereas *Crossosperma* [10] has 4-merous flowers, a completely syncarpous gynoecium, carpels without apical glands, 1 ovule per locule (although it is reported that there are sometimes 2 seeds per locule), stigmas that are broadly peltate with prominent lobes, drupaceous fruit, and seeds that are laterally flattened and winged at the dorsal margin. These substantial differences, perhaps, make the relationship between the two genera less plausible but, given that both genera are highly distinctive and not clearly allied with any other group, the differences could also be a reflection of a long period of divergence between them.

An unusual feature of *Acradenia* is possession of distinctive glandular structures at the apex of each carpel. Among Rutoideae similar structures are seen only in the monotypic South American genus *Pitavia*, which differs from *Acradenia* (like *Crossosperma*) in having indehiscent fruits and 4-merous flowers. *Pitavia* does not show clear affinity to other neotropical groups [2] and unpublished *trn*L-F and *rps*-16 sequences (M. Groppo, Universidade de São Paulo, Brazil; cited by Kubitzki *et al.* [2]) place it among the Australasian Rutoideae, sister to *Flindersia* + *Lunasia*, the only two members of clade E included in the previous phylogeny based on *trn*L-F and *rps*-16 sequences [19]. Given this result, and the similar carpellary glands, it is possible that *Pitavia* and *Acradenia* (and *Crossosperma*) are related. This notion is well worth testing using additional sequences (including further accessions of *Crossosperma*), and could have interesting biogeographic as well as taxonomic implications.

### Evolutionary Patterns in Fruit Morphology and Seed Dispersal

Character reconstructions based on the supertree (Fig. 4) show dehiscent fruits, from which the seeds are ejected at maturity (e.g., Fig. 2D), as ancestral in the Australasian Rutoideae. Derived conditions in the group are: woody capsules with winged seeds (*Flindersia* only; Fig. 2I); samaras (*Pentaceras* only, but paralleled in other groups outside of Australia; Fig. 2J); retention and display of seeds in dehisced fruits (*Melicope* [Fig. 2E], *Geijera*, *Dinosperma* and the pantropical genus *Zanthoxylum*); and indehiscent, fleshy drupes [e.g. Fig. 2F, G, H]. The last two states are inferred to have multiple, independent origins.

Whereas the major sclerophyll lineages in Australasia have dehiscent fruits that forcibly eject the seeds, the greatest diversity of derived fruit types is found in groups that occur primarily in rainforests (compare Figs. 3 and 4). This could relate to differences between these habitats in terms of selective pressures associated with seed dispersal. In Australia these trends mirror those for the flora as a whole, with bird-dispersed seeds (in Rutoideae either encased in fleshy drupes or, as in *Geijera* and *Melicope*, seeds displayed for direct ingestion by seed eating birds) being well represented in rainforests compared with other environments [54]. Likewise, myrmecochory, which is implicated in the dispersal of Rutoideae seeds that are ejected with an attached portion of endocarp (presumed elaiosome, Fig. 2K) [5], [55], [56], as commonly seen in clade C, is generally a feature of drier vegetation types on more infertile soils in Australia [55], [57]. The large winged seeds or fruits of *Flindersia* and *Pentaceras* (Fig. 2I,J), respectively, provide some capacity for air-borne dispersal, at least over short distances. Such morphology is more advantageous in trees such as these, with the chance of dispersal from height [58], [59], than it is for shrubby species of drier vegetation; this is consistent with the observation in Australian subtropical rainforests that wind-assisted propagules are more common in tall trees than in shrubs or small trees [60].

Differences in fruit and seed morphology suggest fleshy drupes have evolved independently in different lineages of Australasian Rutoideae. Coding the drupes of different lineages as equivalent (as done in preparing Fig. 4) is somewhat simplistic, with obvious and substantial differences between groups including those of colour, size, texture and degree of syncarpy. The drupes of *Pitaviaster* are distinct in being almost uniformly 1-carpellate, with the other three carpels aborted and not persisting in fruit [10]. In *Halfordia* (Fig. 2 F) the drupes are 3–5 carpellate and almost completely syncarpous (sometimes with apical septicidal fissures) [7]; in *Dutaillyea* they are four-carpellate and also completely syncarpous [61]. In *Acronychia* (Fig. 2H), *Sarcomelicope*, *Comptonella* and *Picrella* the degree of syncarpy in fruit is variable [9], [62], [63], [64], and within *Acronychia* [9] and *Sarcomelicope* [62] it has been postulated that fruits with more highly fused carpels represent derived states, relative to those that are less strongly fused.

One feature of particular note is the structure of the seed coat in the related drupaceous genera *Acronychia*, *Sarcomelicope*, *Picrella*, *Comptonella* and *Dutaillyea* (all members of clade D; Fig. 4). Related to these taxa is the dehiscent-fruited genus *Melicope*, in which the seeds are retained on the dehisced fruit and show adaptations for dispersal by seed-eating birds (Fig. 2E). The spongy layer of the testa (the sarcotesta) is assumed to be a source of nutrition for seed-eating birds, while the hard inner layer of the testa that it surrounds (the sclerotesta) is proposed to provide structural support for endozoochory [7], [62]. A well-developed sarcotesta, although possibly functionally redundant, is also seen in *Acronychia*, *Sarcomelicope*, *Picrella*, *Comptonella* and *Dutaillyea* [63]. On the basis of this seed feature it has been suggested [62], [63] that the drupes of these genera are derived from *Melicope*-like ancestors. The phylogeny presented here is consistent with this hypothesis, although the relationships are not well-resolved.

### Relationships of *Neoschmidia* and *Halfordia*

The New Caledonian genus *Neoschmidia* has a taxonomic history filled with uncertainty, and a key result of the present study is the resolution of the phylogenetic positions of *Neoschmidia* and *Halfordia* as successive sister taxa to the remainder of clade C. The placement of both taxa is moderately (*Neoschmidia*: BS 78; PP 0.97) to strongly supported (*Halfordia*: BS 89; PP 1.0) by the sequence data. It is also consistent with their possession of 5-merous flowers and alternate, simple leaves, although *Halfordia* differs obviously from most other members of this clade in being a rainforest tree (rather than sclerophyll shrub) with broad glossy, albeit coriaceous leaves and drupaceous, rather than dehiscent, fruits. The placement of *Halfordia* is consistent with that based on more limited taxon sampling of other cpDNA regions (*trn*L-F spacer and *rps*16 intron [19]), but *Neoschmidia* has not been included in previous molecular phylogenies.

Previous works have presented a range of opinions on the relationship of *Neoschmidia* to the Australian members of clade C. The first species was described in 1906 in the genus *Eriostemon* (largely equivalent to *Eriostemon* + *Philotheca* in current classifications [35], [37]), as *E. pallidus* Schltr. That name was an illegitimate later homonym of *E. pallidus* Benth. (F.Muell.) (≡ *Asterolasia pallida* Benth.) and the species did not have a valid name under the International Code of Botanical Nomenclature [65] until erection of the new genus name, *Neoschmidia*, together with description of a segregate species, *N. calycina*, in 2003 [32]. In a morphological cladistic analysis of shrubby, sclerophyllous Australasian genera [5], *Neoschmidia pallida*, as “aff. *Eriostemon*” was resolved as nested within *Eriostemon s. lat*. This was in contrast to treatments of *Eriostemon* and *Philotheca* [37], [46], from which the species was deliberately excluded, with the suggestion [37] that it was part of a lineage comprising *Boronella*, *Myrtopsis*, *Euodia*, *Brombya* and *Medicosma*. It also contrasted with the views of Hartley [32], who noted a resemblance to *Philotheca* and *Eriostemon*, but thought *Neoschmidia* was only distantly related to the those genera and the other shrubby, sclerophyllous taxa of tribe Boronieae (*sensu* [31]). He suggested that its closest relative was *Halfordia*, which he considered “to have no other close relatives” and proposed that *Neoschmidia* should be placed “next to *Halfordia* in tribe Zanthoxleae”. Results here from the *rbc*L + *atp*B analyses do not place these genera as sister taxa, but put them closest to taxa that Hartley would have placed in Boronieae (not Zanthoxyleae); his comments on the relatedness of the two genera were, nonetheless, insightful.

### Biogeographic connections between Australia and New Caledonia

Much recent attention has focused on the history of the biota of New Caledonia and in particular on considering the roles of recent long-distance dispersal versus more ancient vicariance in contributing to the composition of the current flora and flora. The biota of New Caledonia [66] shows high levels of endemism, includes many representatives of gondwanan groups (e.g., Proteaceae, conifers, *Nothofagus*, Myrtaceae including the eucalypt genus *Arillastrum* [67], [68]), and examples of isolated evolutionary lineages (e.g., the flowering plant *Amborella* [69] and the flightless kagu [70]). One explanation for the presence of such taxa in New Caledonia is that their ancestors were present on Zealandia [71], a fragment of continental crust including New Zealand and New Caledonia, when it began rifting from the eastern margin of Australia, beginning c. 85 million years ago (Ma) [72], and that unique lineages such as *Amborella* and the kagu have persisted in isolation on New Caledonia, with extinction of close relatives in other parts of the world. This notion has been challenged by geological evidence [73], [74] that present-day New Caledonia was below sea level for extended periods from the Late Cretaceous (Maastrichtian) through the Palaeocene and Eocene, re-emerging c. 37 Ma. The corollary from some biologists is that the biota reflects colonization since that time [75], potentially through long-distance dispersal, including from Australia. Such a view has received further support from molecular dating studies for a range of plant and animal groups [17], [75], [76], [77], [78], [79]. However, others [66], [80], [81], [82] have argued that the presence of relict lineages and presumed gondwanan groups suggests the presence of at least some land in the New Caledonian region through the period of submergence. Further, some have argued that the geology of the southwest Pacific is complex and that some notions of the tectonic separation of Australian and New Caledonian crustal blocks are overly simplistic [83]. In particular, close terrestrial connections between Australia and the block of continental crust including New Caledonia were potentially more recent than typically assumed. For example, land may have been exposed on the Kenn Plateau until as recently as the late Eocene [83], [84], and on parts of Zealandia including the Lord Howe Rise from the Middle Eocene to Late Oligocene [85], [86], with numerous transient islands (R. Sutherland unpublished data; see http://www.otago.ac.nz/V11-southern-connection/abstracts/#id408), and on the Norfolk Ridge, Reinga Ridge and northernmost Wanganella Ridge in the late Eocene [87]. Subsidence of the New Caledonia Trough may have been as recent as the Oligocene-Miocene [86]. As such, molecular dating studies may be using inappropriate cut-off dates for distinguishing vicariance/dispersal hypotheses, and invoked dispersal distances might not have been as great as suggested by current geography [83].

In addition to geological evidence, data on the phylogenetic relationships of lineages within the New Caledonian biota can give valuable insights into its history. Both patterns of relationships, and levels of sequence divergence in molecular studies, can provide evidence as to which lineages might be recently arrived in New Caledonia (consistent with recolonisation by dispersal after re-emergence) and which might provide evidence for persistence of an older, isolated biota in the New Caledonian region. In the context of the current debate over history of the New Caledonian biota, taxa in the last category are of particular interest. The greater the evidence for the presence of older, isolated lineages in the biota, the stronger the arguments for a general explanation for their presence there (e.g., vicariance, long term persistence of land).

Rutaceae are a useful group to study in the context of New Caledonian biogeography. This is firstly because they are a sizeable proportion of the flora (being one of the five largest families), secondly because some estimated ages for the family [21], [22], [23], [24] could be consistent with a vicariant gondwanan history, and thirdly because within Rutoideae there are multiple lineages with New Caledonian-Australian connections providing, within the one phylogenetic tree, multiple tests of biogeographic connections. The Australia-New Caledonia links include widespread species that occur in both areas (*Halfordia kendack* [43], *Acronychia laevis* [9], *Sarcomelicope simplicifolia* [62], *Geijera salicifolia* [7]), genera that are shared between the two areas but with different species in each (e.g., *Zieria*, *Euodia*, *Flindersia*, *Melicope*, *Medicosma*, *Zanthoxylum*), and the presence of separate but related genera in each of the two areas (e.g., *Acradenia* and *Crossosperma*; *Boronia* and *Boronella*; *Neoschmidia* and *Myrtopsis* both with Australian sclerophyllous taxa).

In the present study we discuss patterns of relationship among taxa, levels of sequence divergence, and estimated divergence ages based on Bayesian relaxed clock dating using a range of fossil calibrations. Although our estimated divergence dates (Table 2) align with those from other studies [23], [24], including a recent study of Rutaceae subf. Spathelioideae [88] also based on Bayesian methods, they differ markedly from much younger divergences estimated for Rutaceae subf. Aurantioideae [17] based on non-parametric rate smoothing [89] and a different range of fossil calibrations (all outside Rutaceae). We recommend caution in interpretation of our divergence estimates, largely because they are influenced by the limited availability of suitable fossil calibrations within Rutaceae. With the exception of two calibrations (Buxaceae and a young fossil calibration for the genus *Skimmia*; Table S3), all fossil-calibrated nodes had lower bounds of 95% highest posterior density intervals corresponding to the enforced minimal age constraints (Table S3), even though the uniform priors used on each of these nodes could have allowed their ages to be much greater. Some trial analyses that included/excluded different calibration points showed that ages for these nodes were much younger in the absence of calibrations; e.g., excluding any calibrations for Sapindales (including Rutaceae) gave an estimated age for the *Ailanthus* stem of 8.5–19.6 (mean 14.3) Ma, compared with a minimum fossil calibration of 52 Ma. This phenomenon has also been discussed in other dating studies of Rutaceae [17], and the implication is that parts of the tree not supported by good calibrations could potentially include substantial underestimates of ages. This is of note because all calibrations in our analyses are somewhat removed from our group of interest, the Australasian Rutoideae (Fig. S2).

**Table 2:** Age estimates for selected New Caledonian/Australian divergences based on Bayesian relaxed clock molecular dating.

Taxon/phylogenetic split	Estimated age (Ma)	Number of nucleotide differences	Postulated dispersal vectors
*Acronychia laevis* 1 (NC)/*A. laevis* 2 (Aus)	(0.0) 0.6 (1.9)	0	Birds
*Sarcomelicope simplicifolia* 3 (NC)/*S. simplicifolia* 2 (LHI)	(1.4) 4.2 (7.6)	0	Birds
*Flindersia fournieri* (NC)/*F. brayleyana* (Aus)	(0.6) 3.9 (8.4)	2	Wind
*Halfordia kendack* 1 (NC)/*H. kendack* 2 and 3 (Aus)	(0.9) 6.6 (14.9)	5	Birds
*Melicope lasioneura* (NC)/*M. elleryana* (Aus)	(0.6) 3.9 (8.0)	5	Birds
*Geijera cauliflora* (NC)/*Geijera* (Aus)	(2.4) 7.0 (12.3)	9–11	Birds
*Euodia teitaensis* (NC)/*E. pubifolia* (Aus)	(4.6) 12.0 (19.7)	13	Ants
*Crossosperma velutina* (NC)/*Acradenia* (Aus)	(5.0) 13.2 (22.1)	15–16	Unknown* (*Acradenia*); birds (*Crossosperma*)
*Boronella* (NC)/*Boronia* (Aus)	(6.1) 13.9 (22.5)	19–21	Ants (*Boronia*)
*Zieria chevalieri* (NC): *Z. madida* (Aus)	(6.5) 11.9 (17.9)	20–24	Ants
*Myrtopsis* stem (NC):	(14.6) 24.3 (33.2)	20–47	Ants
*Neoschmidia* stem (NC)	(31.1) 38.9 (47.0)	27–51	Ants

Estimates include means plus upper and lower bounds of 95% highest posterior density intervals. Numbers of nucleotide differences associated with each divergence are also shown; these are the total number of base differences observed in *rbc*L and *atp*B combined, ignoring missing data and ambiguous base calls. Dispersal vectors are inferred from fruit and seed morphology.

* Seeds are forcibly ejected from the fruits, but have no obvious features for secondary dispersal by ants or other animals (i.e., there is no elaiosome, obvious sarcotesta or pellicle).

Notwithstanding these caveats, the results obtained here, both in terms of patterns of relationship (terminal positions in the tree; Fig. 3), levels of sequence divergence, and estimated divergence ages (Table 2) are consistent, especially for taxa with bird-dispersed or winged seeds, with relatively recent dispersal between Australia and New Caledonia. This is particularly evident, as might be expected from morphological resemblance, between species shared between the two areas, i.e., *Acronychia laevis*, *Halfordia kendack*, and *Sarcomelicope simplicifolia*. It also seems likely for some genera, including *Melicope* and *Flindersia*, with endemic species in both areas.

In some cases, the likely directions of dispersal can be inferred, but for others the data are insufficient to draw firm conclusions. In the case of the widespread *Acronychia laevis*, the only New Caledonian member of *Acronychia*, dispersal from Australia to New Caledonia is inferred. For *Halfordia kendack* the pattern of relationships presented here, invoking the “progression rule” [90], [91], might suggest possible dispersal from New Caledonia to Australia, but our unpublished data, part of an ongoing phylogeographic study of *Halfordia*, clearly support dispersal into New Caledonia, either from Australia or from New Guinea (unsampled). *Sarcomelicope* (9 species), with the exception of the widespread species *S. simplicifolia*, is endemic to New Caledonia; this could suggest a direction of dispersal in *S. simplicifolia* from New Caledonia to Australia, however, sampling here does not demonstrate monophyly of the genus and does not include any representative of the genus from mainland Australia (only Lord Howe Island; Table S1), so firm conclusions cannot be drawn without further sampling and better resolution of relationships. The same is true for the large genus *Melicope* (233 species) in which the five New Caledonian species (all endemic) are placed in sect. *Pelea* [22] and thus likely to be most closely related not to the three Australian (sect. *Lepta*) or one New Zealand (sect. *Melicope*) species sampled here, but rather to unsampled species of sect. *Pelea*, which occur chiefly in Malesia (especially New Guinea) and the Pacific [22], [30]. For *Flindersia*, with just one phylogenetically nested species in New Caledonia (*F. fournieri*; Fig. 3), dispersal into New Caledonia from either Australia or New Guinea (unsampled) is consistent with the data presented here and with previous analysis of ITS sequences [18].

In contrast to the shallow Australia–New Caledonia divergences in some groups, there are other divergences that are deeper in the tree (Fig. 3), involve more substantial sequence differences and older divergence age estimates (Table 2). These include the *Crossosperma*/*Acradenia* lineage (discussed above), *Boronia*/*Boronella*, *Zieria*, *Myrtopsis* and *Neoschmidia*. Apart from *Crossosperma*/*Acradenia*, these include lineages that are presumed, on the basis of seed morphology, to be ant dispersed and not as amenable to long-distance dispersal as plants with adaptations for bird dispersal. For *Zieria* and *Boronia*/*Boronella* the Australia–New Caledonia sequence divergences are substantial. However, the sampling of these genera is currently insufficient to give an accurate picture of either the pattern of relationship, or level of sequence divergence; i.e., it is likely that sampling does not include the Australian species of *Zieria* (59 species) and *Boronia* (c. 148 species) most closely related to those of New Caledonia. Likewise, *Myrtopsis* is placed here in a large clade (representing 15 genera and c. 183 species) that is only sparsely sampled, and lack of phylogenetic resolution makes it unclear how *Myrtopsis* is related to other members of this clade.

Of particular interest in the context of biogeography, as well as in terms of phylogeny/classification (see above), is the position of the endemic New Caledonian genus *Neoschmidia* (2 species) as sister to the other members of clade C, with good support (BS 78; PP 0.97). This is the deepest Australia/New Caledonia divergence in the tree. With the exception of *Halfordia* and *Myrtopsis*, the sister lineage of *Neoschmidia* includes mostly sclerophyllous Australian genera (15 genera and c. 182 species, plus one unsampled species of *Leionema* from New Zealand). Given their patterns of distribution and species richness, these Australian genera most likely have an extended Cenozoic history [5], [25], [92]. In particular, a number of the genera and some of the sections are disjunctly distributed between southeast and southwest Australia with endemic species in both areas, e.g., *Asterolasia*, *Crowea*, *Phebalium*, *Philotheca* sect. *Corynonema*, *Philotheca* sect. *Erionema*, *Philotheca* sect. *Philotheca*, *Nematolepis*. Such disjunctions are common in the Australian flora and are generally attributed to edaphic and climatic changes subsequent to marine incursion over the Great Australian Bight, at least in the mid-Miocene, c. 16–14 Ma, if not to similar events in the Eocene, c. 35 Ma [93], [94], [95]. A vicariance explanation for these disjunctions [90], [93] would suggest that multiple lineages had diverged prior to the formation of one or more of these barriers across southern Australia, and that divergence of *Neoschmidia* (and *Halfordia*) from this group is logically an older event.

The estimated age of divergence of *Neoschmidia* from other members of clade C is 31.1– 47.0 (mean 38.9) Ma. This is a substantial divergence, consistent with inferences (above) regarding the age of Australian relatives, but implications for Australia–New Caledonia biogeographic history are not clear. The estimate overlaps with the re-emergence of New Caledonia c. 37 Ma [73], [74] and could be consistent with over-water dispersal to New Caledonia since that time. Alternatively, the upper range of the estimate pre-dates this time, coinciding with the presence of exposed land between Australia and New Caledonia [83], [84], [85], [86], allowing some possibility for terrestrial connection to the greater New Caledonian area or, at least, for smaller dispersal distances. It also coincides with a period when large parts of Zealandia subsided [83], [84], [85], [86], [96], [97], possibly initiating vicariant divergence. Given potential limits to the dispersal ability of these plants (presumed ant-dispersal), imprecise knowledge of geological history, and the fact that our age estimates, as discussed above, could be underestimates, the data presented here are consistent with a complex history involving vicariance and do not provide strong support for favouring a hypothesis of long distance dispersal in the history of *Neoschmidia*.

Of all of the taxa in our dataset, it is *Neoschmidia* along with *Myrtopsis*, *Boronia*/*Boronella*, *Zieria* and *Crossosperma*/*Acradenia* that are the most likely candidates for representing old, vicariant links between Australia and the New Caledonian region. Further investigation of their relationships and sequence divergences would be worthwhile, especially additional sampling of related taxa in clades C and D (Fig. 3) and use of additional molecular markers (non-coding in particular) for phylogenetic and dating studies.

### Concluding Remarks

Molecular phylogenetic analyses of Rutaceae world-wide, and our comprehensive sampling of subfamily Rutoideae and Australasian genera, show that major clades do not align with current subfamilial and tribal classification, which thus require significant revision. Within Rutoideae, Australasian lineages fall into two main clades, each with early divergences between sclerophyll and rainforest taxa. Rainforest taxa have independently evolved fleshy fruits or prominent display of seeds in the canopy a number of times; some of these taxa have relatively wide geographic distributions in eastern Australia, Malesia and the South West Pacific and low sequence divergence, which suggest that these traits favour vertebrate dispersal of fruit and seed. Sclerophyll taxa that have relatively deep clade divergences between geographic regions, as well as drier, dehiscent fruit (an ancestral trait) and limited seed dispersal ability (e.g., ant dispersal) suggest a biogeographic history of vicariance. Biogeographic connections among Rutaceae between Australia and New Caledonia are potentially explained by both processes of long distance dispersal over water barriers and older vicariance. This study highlights those taxa that should be sampled in greater detail before major taxonomic changes are formalized and for testing our biogeographic hypotheses. Further analyses based on nuclear DNA markers would be worthwhile, to test for congruence with relationships inferred here from chloroplast DNA sequences.

## Materials and Methods

### DNA Isolation, Amplification and Sequencing

Plant material was obtained from field collections and in a few cases from existing herbarium specimens. Collecting permits were provided by Parks Victoria, the Western Australian Department of Environment and Conservation, New South Wales Parks and Wildlife Service, Queensland Environmental Protection Agency, Australian National Botanic Gardens, and conservation authorities of the North and South Provinces of New Caledonia (DDEE, Direction du Développement Économique et de l’Environnement; DENV, Direction de l'Environnement). Details of sampling locations and voucher specimens are given in Table S1. DNA was isolated from leaf tissue (dried in silica gel for new field collections) using the DNeasy® Plant Mini Kit (Qiagen) following the manufacturer’s instructions, with a final elution volume of 100 µL. The *rbc*L region was most commonly amplified using the primers RUTrbcL1F (ATGTCACCACAAACAGAGACTAAAGC) and rbcL1343R (GCCTCCCGGATAATTTCATT); in a few cases where PCR amplification with these primers was unsuccessful the region was amplified in two overlapping fragments using: RUTrbcL1F paired with RUTrbcL724R (TCGCATGTCCCTGCAGTAGC); RUTrbcL636F (GCGTTGGAGGGACCGTTTCT) paired with rbcL1343R. The *atp*B gene was amplified in two overlapping fragments using: RUTatpB2 (TATGAGAATAAATCCTACTACTTCC) paired with RUT766R (TAACATCTCGGAAATATTCYGCCAT); 611F (AACGTACTCGTGAAGGAAATGATCT) paired with 1494R (TCAGTACACAAAGATTTAAGGTCAT) [98]. PCR mixtures for *rbc*L amplification included 0.4 µM of each primer, 200 µM of each dNTP, 1–1.2 µl of DNA extract, 0.625 U HotStarTaq DNA polymerase and its 10 X PCR buffer (QIAGEN, Germany; including a final Mg^2+^ concentration of 1.5 mM), and were made to 25 µL with ultrapure water. PCR mixtures for *atp*B included the same ingredients but with inclusion of 1 µL DMSO. All amplifications were performed using a touchdown protocol with the following cycling conditions: 95°C for 15 min; 5 cycles of 94°C for 45 sec, 60°C for 45 sec (with a decrease of 2°C in each subsequent cycle), and 72°C for 1 min; 30 cycles of 94°C for 45 sec, 50°C for 45 sec and 72°C for 1 min; 72°C for 5 min after the last cycle. PCR products were purified using a QIAquick PCR Purification Kit (QIAGEN, Germany), QIAquick Gel Extraction Kit (QIAGEN, Germany) or PureLink Kit (Invitrogen, Australia). PCR amplifications of *rbc*L for members of the genus *Geijera* frequently yielded two amplicons: one of the expected size and a much shorter product. The shorter amplicon was shown by sequencing to differ from the expected product by an 831 bp deletion; this truncated *rbc*L sequence potentially represents a pseudogene and was excluded from further analysis. PCR products were directly sequenced using the amplification primers (with rbcL724R and rbcL636F occasionally used as internal sequencing primers for *rbc*L) and the ABI Prism® BigDye Terminator v3.1 Cycle Sequencing Ready Reaction Kit (Applied Biosystems, U.S.A.). Sequences were analysed on an ABI 3730x1 96-capillary automated DNA sequencer, at the Australian Genome Research Facility, Brisbane or Melbourne.

### Sequence Editing and Alignment

Contiguous sequences were assembled with Sequencher *v.* 3.0 (Gene Codes Corporation, Ann Arbor, MI, USA) and manually aligned with Se-Al Sequence Alignment Editor *v.* 2.0 [99]; there were no indels, so alignments were unproblematic. Individual sequences are available from GenBank (Table S1)

### Phylogenetic Analyses

Sequences were analysed using maximum parsimony (MP) with PAUP* 4.0 beta 10 [100] and Bayesian inference (BI) using MrBayes v.3.1.2 [101]. MP analyses produced many equally parsimonious trees, so the following search strategy was used. The initial heuristic tree search (based on a CLOSEST addition sequence and TBR branch swapping, with all characters equally weighted and gaps, present at the ends of the alignment, treated as missing data) was stopped at 40,000 trees and a strict consensus tree was calculated. A second analysis searched only for trees that were not consistent with this strict consensus and used 1,000 random addition sequences, each followed by TBR branch swapping, aborting each replicate when 5,000 trees (of a length exceeding those of the first analysis) were obtained; the purpose of this was to ascertain whether the strict consensus was likely to represent adequately the full set of equally most parsimonious trees, even though these were not all obtained. The first time this strategy was used, trees of the same length, inconsistent with the initial consensus were found. A further consensus, including the topology of these trees was obtained, and the second step of the analysis was repeated. Bootstrap analysis was carried out using the heuristic search option and 1000 replicates with a MAXTREES of 2,000 for each replicate.

BI analyses used GTR+Γ+I model of sequence evolution for both *atp*B and *rbc*L (with parameters unlinked between partitions), because this was the preferred model for both datasets using the Akaike Information Criterion as implemented in MrModeltest 2.3 [102]. The analysis used the default settings of MrBayes and included two runs of four chains, each run for 4.1 million generations. Trees were sampled every 500 generations and a majority rule consensus was computed (with trees from the first 500,000 generations discarded as burn-in). That the two runs had converged on a stationary distribution, and that the burn-in period was adequate, was judged by comparing the distribution of likelihood values in Tracer v.1.5 [103], and the standard deviation of split frequencies (which were < 0.01 at the end of the runs).

The results of this study were combined with those of previous molecular phylogenetic studies to produce a summary (supertree) of relationships in the family Rutaceae. DNA sequence data from published studies of Rutaceae, based on different combinations of markers, are not directly amenable to combined analysis, so a supertree approach, integrating tree topologies from different studies was more feasible. Published phylogenies for Rutaceae are overwhelmingly congruent, largely complementary (focusing on different subgroups), and the backbone of the tree (e.g., as shown in Fig 1) is well supported by multiple studies [16], [19] based on different genes. Thus, as a preliminary step toward summarizing relationships, rather than using more numerical approaches to supertree construction [104], [105], [106], [107], we have taken an intuitive (“consensus” or “indirect”) approach [107], simply combining in a manual fashion the compatible components of published studies, mostly using genera (where monophyletic) as terminal taxa. The backbone of the supertree comes from the most comprehensive family-wide studies to date [16], [19], which are consistent with the results of other, more narrowly-focused studies. The most detailed studies of subf. Aurantioideae [108], subf. Cneoroideae [109], tribe Ruteae [110] and tribe Diosmeae [111], provided topology of the supertree for those groups, and trees from other studies are used to infer the placement of the genera *Boenninghausenia* [110], *Chloroxylon* [16], [19], *Fagaropsis* [112], *Microcybe* [25], *Muiriantha* [25], *Orixa* [29], [110], *Platydesma* [30], *Rhadinothamnus* [25], *Tetradium* [29], [112], *Thamnosma* [110], [113], *Toddalia* [29], [108]. Where there was any possible ambiguity over the placement of a taxon, e.g. because of sampling differences between studies, a conservative approach was taken, giving the minimal resolution consistent with the input trees. *Chloroxylon* (3 species from Madagascar, southern India and Sri Lanka) was placed in clade R1 (Rutoideae s. str, tribe Ruteae) on the supertree on the basis of analyses of *rbc*L + *atp*B [16], and *trn*L-F + *rps*16 [19] sequences, all derived from the same genomic DNA. This placement has been questioned on morphological grounds [2], [19], [22] as *Chloroxylon* has traditionally been placed with *Flindersia* in tribe Flinderseae (clade E here). A recent phylogenetic analysis of Ruteae [110] did not include, or even mention, *Chloroxylon*. The placement of *Chloroxylon* in a conservative position in that group in the supertree results in some collapse of resolution when compared with that study.

Selected morphological characters were optimized on the supertree in MacClade [114] using parsimony and the option to trace “unambiguous changes only”. Character data were scored from the literature, including relevant revisions/monographs [6], [9], [10], [22], [32], [34], [39], [45], [53], [62], [115], [116], field guides [49], [117], flora treatments [118] and other works [2], and from personal observations. The definition of character states was mostly straightforward, as outlined on Figs 4–5. In terms of leaf complexity, compound leaves were grouped with those considered in the literature to be unifoliate (as opposed to simple), usually on the grounds that the petiole is distally swollen or that there is jointing between leaflet lamina and petiole.

### Molecular Divergence Dating

Bayesian relaxed clock molecular dating was used to estimate the timing of phylogenetic divergences in the Australasian Rutoideae, with a particular focus on divergences between New Caledonian and Australian taxa, in order to provide insight into their biogeographic history. Dating analyses were based on an expanded set of *rbc*L and *atp*B sequences for an additional 94 taxa representing major groups of core eudicots (Table S2). This allowed inclusion of a range of fossil calibrations external to Rutaceae (for which there is a paucity of well-dated fossils that can be reliably assigned to nodes on the phylogenetic tree) and to avoid the use of any secondary calibrations (constraints based on estimates from previous dating studies), which are often problematic [119]. In total 15 fossil constraints were used (Table S3), including three from Rutaceae, one from the closely related family Simaroubaceae and 11 from across the major clades of eudicots. These calibrations have been used and discussed in detail in other dating studies [88], [119], [120], [121], [122]. The root of the tree, divergence of Ranunculales from other core eudicots was fixed at 125 Ma, based on the appearance of tricolpate pollen [123], [124]. Dating analyses were performed in Beast v.1.7.5 [125] using the GTR+Г+I evolutionary model (unlinked for the two data partitions), birth-death model of speciation, and a relaxed clock with uncorrelated lognormal distribution. Two separate analyses were run for 100 million generations. Tracer v. 1.5 (http://beast.bio.ed.ac.uk/Tracer) was used to check for convergence between runs, the suitability of the burn-in fraction, the effective sample size for parameters (all >200 in each run), and to summarise age estimates and confidence intervals for nodes of interest, using combined data from the two runs. Data from the two runs were also combined using Logcombiner 1.7.5 and used to produce a maximum clade credibility tree in TreeAnnotator 1.7.5 [125]. In order to assess the informativeness of fossil constraints (the extent to which minimum bounds affected node heights), all fossil calibrations, except for the root, were set with uniform priors between a hard minimum bound and the root height (125 Ma).

## Supporting Information

Figure S1 **One of the shortest trees (chosen at random) produced by MP analysis of combined** ***rbc*** **L and** ***atp*** **B sequences.** The tree is drawn as a phylogram with branch lengths proportional to inferred sequence changes. Major clades of Australasian Rutoideae (A-E) are labelled as on Fig. 3.
(PDF)

Figure S2 **Maximum clade credibility tree produced by relaxed-clock molecular dating analysis in Beast** (spread over two pages). Node heights indicate mean age estimates. The time scale is in millions of years. **A**, basal portion of tree with nodes calibrated by fossil constraints indicated by numbers in circles (numbers match details in Table S3). **B**, portion of tree including Australasian Rutaceae. Blue error bars indicate the 95% highest posterior density for node heights. Stars indicate nodes representing Australian-New Caledonian divergences, as described in Table 2.
(PDF)

Table S1 **Details of sequences (GenBank numbers) and samples used in this study.**
(DOC)

Table S2 **Details of additional sequences (GenBank numbers) included in molecular dating analyses**.
(DOCX)

Table S3 **Fossil calibrations used in molecular dating analyses.**
(DOCX)
