# Supplementary figures and images for: Major Clades of Australasian Rutoideae (Rutaceae) Based on rbcL and atpB Sequences
Source: PLoS One. 2013 Aug 13;8(8):e72493. doi: 10.1371/journal.pone.0072493 (PMC3742607; doi:10.1371/journal.pone.0072493)

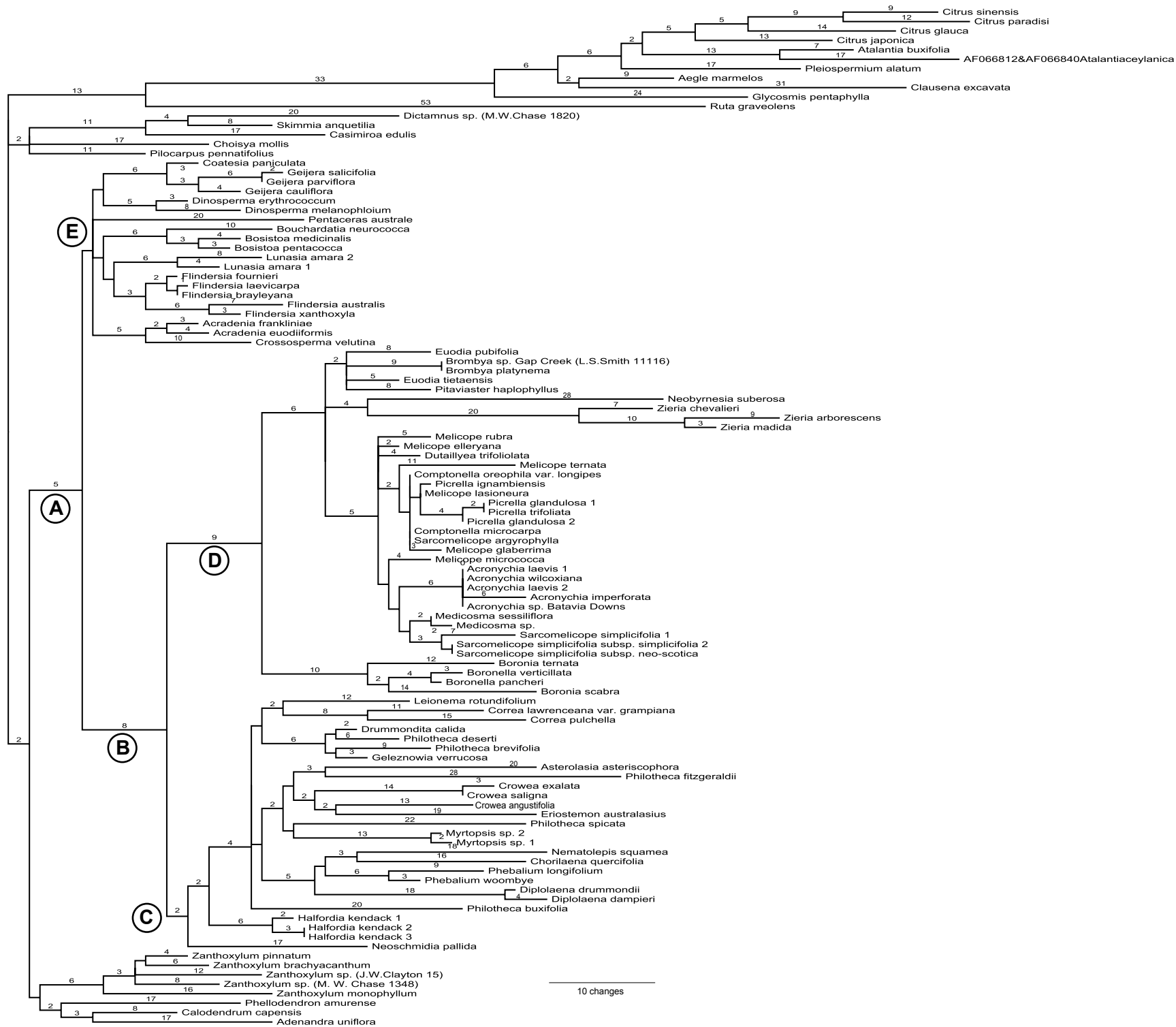

Supplement: Figure S1 — One of the shortest trees (chosen at random) produced by MP analysis of combined rbc L and atp B sequences. The tree is drawn as a phylogram with branch lengths proportional to inferred sequence changes. Major clades of Australasian Rutoideae (A-E) are labelled as on Fig. 3. (PDF) [file pone.0072493.s001.pdf]

A

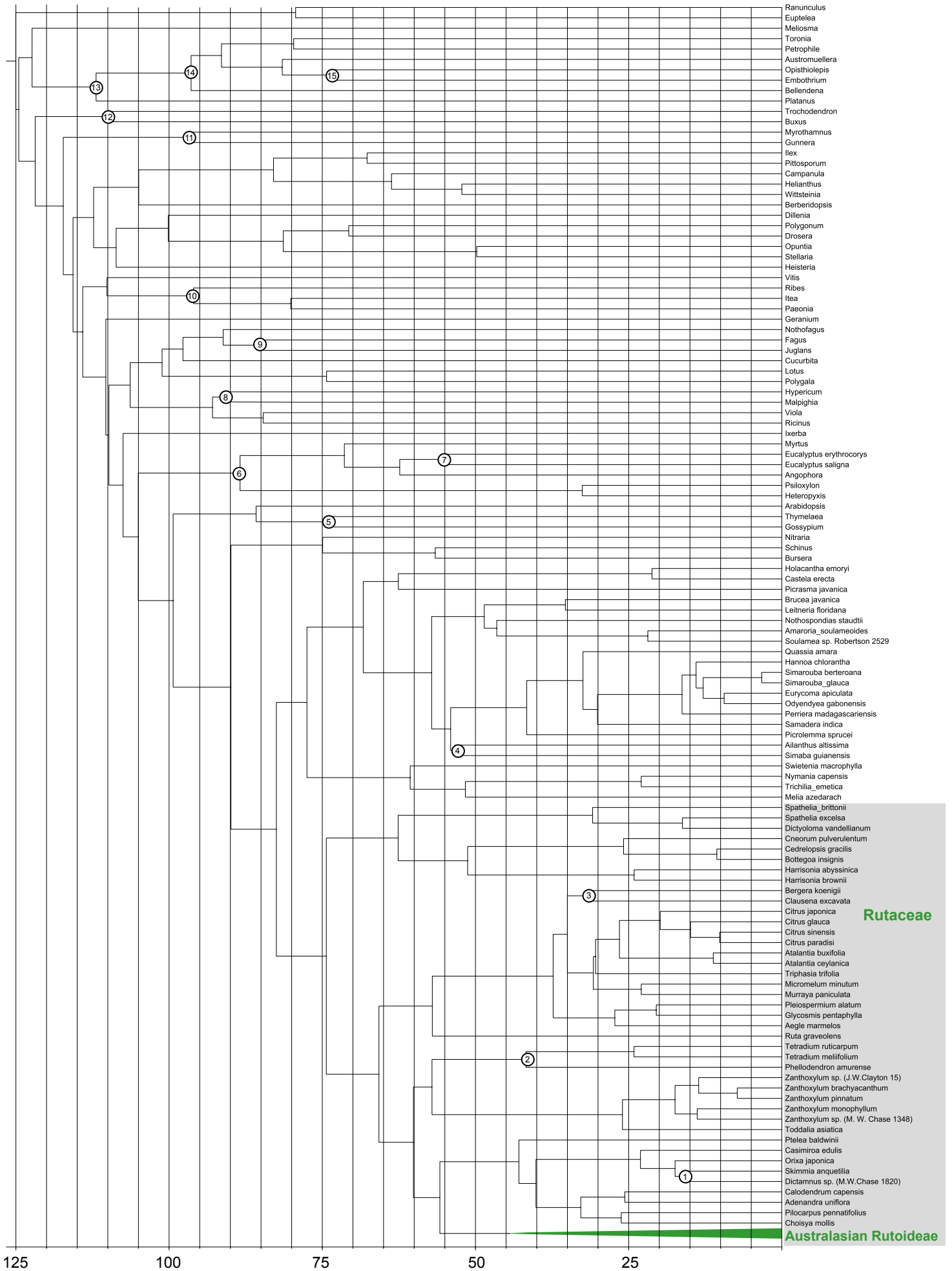

B

Joins rest of tree.  
See Fig. S2A

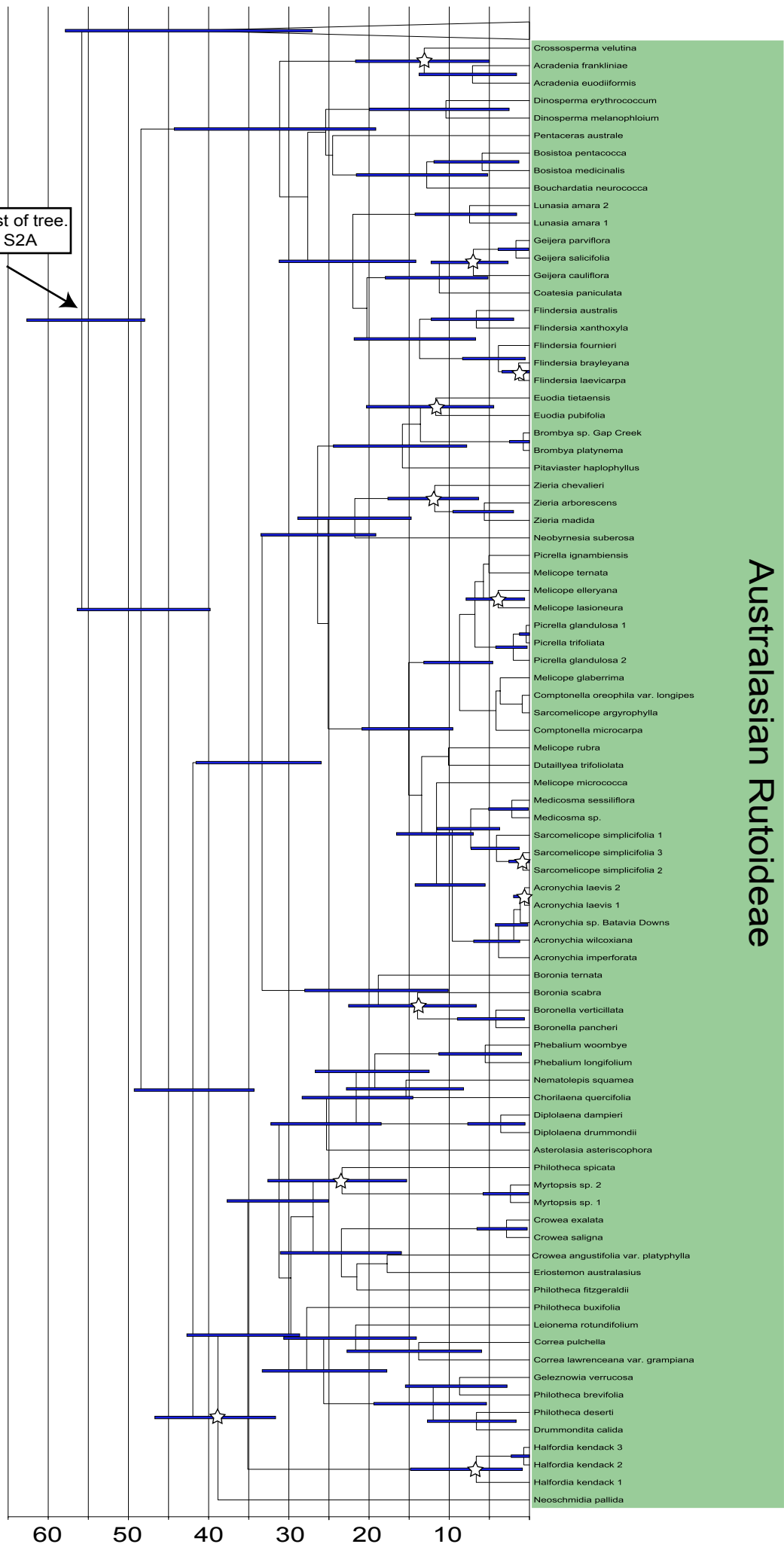

Supplement: Figure S2 — Maximum clade credibility tree produced by relaxed-clock molecular dating analysis in Beast (spread over two pages). Node heights indicate mean age estimates. The time scale is in millions of years. A, basal portion of tree with nodes calibrated by fossil constraints indicated by numbers in circles (numbers match details in Table S3). B, portion of tree including Australasian Rutaceae. Blue error bars indicate the 95% highest posterior density for node heights. Stars indicate nodes representing Australian-New Caledonian divergences, as described in Table 2. (PDF) [file pone.0072493.s002.pdf]
