# Supplementary material for: Major Clades of Australasian Rutoideae (Rutaceae) Based on rbcL and atpB Sequences
Source: PLoS One. 2013 Aug 13;8(8):e72493. doi: 10.1371/journal.pone.0072493 (PMC3742607; doi:10.1371/journal.pone.0072493)
Supplement: Table S1 — Details of sequences (GenBank numbers) and samples used in this study. (DOC) [file pone.0072493.s003.doc]

## Table S1 Details of sequences (GenBank numbers) and samples used in this study.

| **Species** | ***rbc*L** | ***atp*B** | **Voucher details or reference** |
| --- | --- | --- | --- |
| *Acradenia* *euodiiformis* (F.Muell.) T.G.Hartley | JN987069 | JN986991 | Springbrook, Qld, P.I. Forster 34177, BRI |
| *Acradenia* *frankliniae* Kippist | JN987070 | JN986992 | Cultivated Royal Botanic Gardens, Melbourne, M.J. Bayly 1964, MELU 105871 |
| *Acronychia* *imperforata* F.Muell. | JN987071 | JN986993 | Deepwater National Park, Qld, P.I. Forster 33333, BRI |
| *Acronychia* *laevis* J.R.Forst. & G.Forst **1** | JN987073 | JN987064 | Grongah National Park, Qld, P.I. Forster 33410, BRI |
| *Acronychia* *laevis* J.R.Forst. & G.Forst **2** | JN987072 | JN986994 | Boulinda, New Caledonia, M.J. Bayly 2084, MEL, BRI, NOU |
| *Acronychia* sp. Batavia Downs (J.R.Clarkson 8511) | JN987074 | JN986995 | Cultivated Tolga, ex Chester River, Silver Plains, Qld, Sankowsky 1999, AQ0777516 |
| *Acronychia* *wilcoxiana* (F.Muell.) T.G.Hartley | JN987075 | JN986996 | Eumundi, Qld, P.I. Forster 33502, BRI |
| *Adenandra* *uniflora* Willd. | AF066803 | AF066832 | Chase *et al.* (1999) |
| *Aegle* *marmelos* (L.) Correa ex Roxb. | AF066811 | AF066839 | Chase *et al.* (1999) |
| *Asterolasia* *asteriscophora* (F.Muell.) Druce subsp. *asteriscophora* | JN987076 | JN986990 | Mt Buffalo National Park, Vic, A.V. Kellow 223, MELU D103547 |
| *Atalantia ceylanica* **(Arn.) Oliver** | AF066812 | AF066840 | Chase *et al.* (1999) |
| *Atalantia* *buxifolia* (Poir.) Oliv. | AF066806[[1]](#footnote-2) | AF066835Error: Reference source not found | Chase *et al.* (1999) |
| *Boronella pancheri* Baill. | JN987078 | JN986998 | Creek Pernod, New Caledonia, M.J. Bayly 2046, MEL, BRI, NOU |
| *Boronella verticillata* Baill. ex Guillaumin | JN987077 | JN986997 | Creek Pernod, New Caledonia, M.J. Bayly 2047, MEL, BRI, NOU |
| *Boronia* *scabra* Lindl. subsp. scabra (WA) | JN987079 | JN986999 | South Coast Highway, WA, M.J. Bayly 1946, MEL |
| *Boronia* *ternata* Endl. | JN987080 | JN987000 | Boorabbin National Park, WA, M.J. Bayly 1931, MEL |
| *Bosistoa medicinalis* (F.Muell.) T.G.Hartley | JN987081 | JN987001 | Garraway Creek, Qld, Sankowsky 2942, MELU 105885 |
| *Bosistoa* *pentacocca* (F.Muell.) Baill. | JN987082 | JN987002 | Cultivated Tolga, ex Granite Creek, Sankowsky 2950, MELU 105884 |
| *Bouchardatia* *neurococca* (F.Muell.) Baill. | JN987083 | JN987003 | Cultivated Tolga, ex Goodnight Scrub National Park, Qld, Sankowsky 2949, MELU 105886 |
| *Brombya* *platynema* F.Muell. | JN987084 | JN987004 | Daintree Discovery Centre, Qld, P. Green s.n., MELU 105878 |
| *Brombya* sp. Gap Creek (L.S.Smith 11116) | JN987085 | JN987005 | Cultivated Tolga, ex Mt Finnegan, Qld, Sankowsky 1389, BRI AQ0549743 |
| *Calodendrum* *capensis* (L.f.) Thunb. | AF066805 | AF066834 | Chase *et al.* (1999) |
| *Casimiroa* *edulis* La Llave | AF066808 | AF066837 | Chase *et al.* (1999) |
| *Choisya* *mollis* Standley | AF066800 | AF066829 | Chase *et al.* (1999) |
| *Chorilaena* *quercifolia* Endl. | AF066810 | AF066838 | Chase *et al.* (1999) |
| *Citrus* *glauca* (Lindl.) Burkill | AF066819[[2]](#footnote-3) | AF066847Error: Reference source not found | Chase *et al.* (1999) |
| *Citrus japonica* Thunb. | AF066799[[3]](#footnote-4) | AF066827Error: Reference source not found | Chase *et al.* (1999) |
| *Citrus* *paradisi* Macfad. | AJ238407 | AJ238408 | Chase *et al.* (1999) |
| *Citrus sinensis* (L.) Osbeck | DQ864733 | DQ864733 | Bausher *et al.* (2006) |
| *Clausena* *excavata* Burm. f. | AF066813 | AF066841 | Chase *et al.* (1999) |
| *Coatesia* *paniculata* F.Muell. | JN987086 | JN987006 | Jack Smith's Scrub Conservation Park, Qld, P.I. Forster 32991, BRI |
| *Comptonella microcarpa* (Perkins) T.G.Hartley | JN987087 | JN987007 | Koniambo, New Caledonia, M.J. Bayly 2089, NOU, MEL, BRI |
| *Comptonella oreophila var. longipes* (Guillaumin) T.G.Hartley | JN987088 | JN987008 | Plateau de Dogny, New Caledonia, M.J. Bayly 2078, MEL, BRI, NOU |
| *Correa* *lawrenceana* var. *grampiana* Paul G.Wilson | JN987089 | JN987009 | Grampians National Park, Vic., M.J. Bayly 1988, MEL |
| *Correa* *pulchella* Mackay ex Sweet | AF066816 | AF066844 | Chase *et al.* (1999) |
| *Crossosperma velutina* (Guillaumin) T.G.Hartley | JN987090 | JN987065 | Forêt des Electriques, sentier à proximité du Grand Houp, New Caledonia, Barrabé 417, NOU |
| *Crowea* *angustifolia* var. *platyphylla* Benth. | JN987091 | JN987066 | Shannon National Park, WA, M.J. Bayly 1953, MEL |
| *Crowea* *exalata* F.Muell. subsp. *exalata* | JN987092 | JN987010 | W Tree Falls, Vic., D.J. Ohlsen s.n., MELU |
| *Crowea* *saligna* Andrews | JN987093 | JN987011 | Waterfall, Sydney, NSW, D.J. Ohlsen s.n., MELU |
| *Dictamnus* sp. (M.W.Chase 1820, K) | AF066801 | AF066830 | Chase *et al.* (1999) |
| *Dinosperma* *erythrococcum* (F.Muell.) T.G.Hartley | JN987094 | JN987012 | Grongah National Park, Qld, P.I. Forster 33407, BRI |
| *Dinosperma melanophloium* (C.T.White) T.G.Hartley | JN987095 | JN987013 | CSIRO arboretum, Atherton, ex Dinden National Park, Qld, M.J. Bayly 1887, MELU 105897 |
| *Diplolaena* *dampieri* Desf. | AF066807 | AF066836 | Chase *et al.* (1999) |
| *Diplolaena drummondii* (Benth.) Ostenf. | JN987096 | JN987014 | Wellington National Park, Qld, M.J. Bayly 1956, MEL |
| *Drummondita calida* (F.Muell.) Paul G.Wilson | JN987097 | JN987015 | Bulleringa National Park, Qld, P.I. Forster 22556, BRI AQ0605109 |
| *Dutaillyea trifoliolata* Baill. | JN987098 | JN987067 | Mandjelia, New Caledonia, M.J. Bayly 2094, MEL, BRI, NOU |
| *Eriostemon* *australasius* Pers. | JN987099 | JN987016 | Cultivated Rosanna, Vic., M.J. Bayly 1869, MELU 105864 |
| *Euodia* *pubifolia* T.G.Hartley | JN987100 | JN987017 | Cultivated, ex Daintree National Park, Qld, from P.I. Forster 25751, BRI AQ0607159 |
| *Euodia tietaensis* (Guillaumin) T.G.Hartley | JN987101 | JN987018 | Massif du Koniambo, vallée de la Tiave, New Caledonia, J. Munzinger 4530, NOU |
| *Flindersia* *australis* R.Br. | JN987102 | JN987019 | Coolabunia, Qld, P.I. Forster 28937, BRI |
| *Flindersia* *brayleyana* F.Muell. | JN987103 | JN987020 | Davies Creek National Park, Qld, P. Green s.n., MELU 105881 |
| *Flindersia fournieri* Pancher & Sebert | JN987104 | JN987021 | Kuébini river, pont près de l'embouchure, New Caledonia, J. Munzinger 4842, NOU |
| *Flindersia* *laevicarpa* C.T.White & W.D.Francis | JN987105 | JN987022 | CSIRO arboretum, Atherton, ex near Danbulla National Park, Qld, M.J. Bayly 1886, MELU 105898 |
| *Flindersia* *xanthoxyla* (A.Cunn. ex Hook.) Domin | JN987106 | JN987023 | Coolabunia, Qld, P.I. Forster 32512, BRI AQ0618506 |
| *Geijera cauliflora* Baill. | JN987107 | JN987024 | Pindai, New Caledonia, M.J. Bayly 2086, MEL, BRI, NOU |
| *Geijera* *parviflora* Lindl. | JN987108 | JN987025 | Ban Ban Springs, Qld, P.I. Forster 31159, BRI |
| *Geijera* *salicifolia* Schott | JN987109 | JN987026 | Cultivated Tolga, ex Toowoomba, Qld, Sankowsky 2941, arb 1288, MELU 105890 |
| *Geleznowia* *verrucosa* Turcz. | JN987110 | JN987027 | Tathra National Park, WA, M.J. Bayly 1910, MEL |
| *Glycosmis* *pentaphylla* (Retz.) DC. | AF066820 | AF066849 | Chase *et al.* (1999) |
| *Halfordia* *kendack* (Montrouz.) Guillaumin **2** | JN987111 | JN987028 | Cultivated Tolga, ex Cape York, Qld, Sankowsky 2959, MELU |
| *Halfordia* *kendack* (Montrouz.) Guillaumin **1** | JN987112 | JN987029 | Koniambo, New Caledonia, M.J. Bayly 2066, MEL, BRI, NOU |
| *Halfordia* *kendack* (Montrouz.) Guillaumin **3** | JN987113 | JN987030 | Eurimbula, Qld, P.I. Forster 33322, BRI |
| *Leionema* *rotundifolium* (Endl.) Paul G.Wilson | JN987114 | JN987031 | Girraween National Park, Qld, P.I. Forster 34469, BRI |
| *Lunasia* *amara* Blanco **2** | AF066814 | AF066842 | Chase *et al.* (1999) |
| *Lunasia* *amara* Blanco **1** | JN987115 | JN987032 | Cultivted Tolga, ex Iron Range, Qld, Sankowsky 2955, MELU 105891 |
| *Medicosma* *sessiliflora* (C.T.White) T.G.Hartley | JN987116 | JN987033 | Cultivated Tolga, ex Parrot Creek, Shiptons Flat, Qld, Sankowsky 1572, BRI AQ0654312 |
| *Medicosma* sp. "Boonjee" (variant of *M*. *glandulosa* T.G.Hartley *sens. lat*.) | JN987117 | JN987034 | Cultivated Tolga, ex Boonjee, Qld, Sankowsky 1559, BRI AQ0654307 |
| *Melicope* *elleryana* (F.Muell.) T.G.Hartley | JN987118 | JN987035 | Shelburne Bay (Nixon) Homestead, Qld, P.I. Forster 34003, BRI |
| *Melicope glaberrima* Guillaumin | JN987119 | JN987036 | Plateau de Dogny, New Caledonia, M.J. Bayly 2073, MEL, BRI, NOU |
| *Melicope lasioneura* Baill. ex Guillaumin | JN987120 | JN987037 | Réserve du Pic du Grand Kaori, New Caledonia, J. Munzinger 4490, NOU |
| *Melicope* *micrococca* (F.Muell.) T.G.Hartley | JN987121 | JN987038 | D'Aguilar Range, Qld, P.I. Forster 33510, BRI |
| *Melicope* *rubra* (Lauterb. & K.Schum.) T.G.Hartley | JN987122 | JN987039 | Cultivated Tolga, ex Lankelly Creek, Qld, P.I. Forster 15262, BRI |
| *Melicope ternata* J.R. Forst. & G. Forst. | AF116271 | AF066826 | Chase *et al.* (1999) |
| *Myrtopsis* sp. 1 | JN987123 | JN987040 | Plum, New Caledonia, M.J. Bayly 2048, MEL, BRI, NOU |
| *Myrtopsis* sp. 2 | JN987068 | JN987041 | Grand Kaori, New Caledonia, M.J. Bayly 2060, MEL, BRI, NOU |
| *Nematolepis* *squamea* (Labill.) Paul G.Wilson subsp. *squamea* | JN987124 | JN987042 | Wooyung, near Billinudgel, NSW, P.I. Forster 34811, BRI |
| *Neobyrnesia* *suberosa* J.A.Armstr. | JN987125 | JN987043 | Kakadu National Park, NT, M.J. Bayly 1904, MEL |
| *Neoschmidia pallida* T.G.Hartley | JN987126 | JN987044 | Cultivated Royal Botanic Gardens Sydney, ex Mt Dore, New Caledonia, P.H. Weston 3303, NSW |
| *Pentaceras* *australe* (F.Muell.) Benth. | JN987127 | JN987045 | D'Aguilar Range, Qld, P.I. Forster 30445, BRI AQ0609828 |
| *Phebalium* *longifolium* S.T. Blake | JN987128 | JN987046 | Mount Baldy State Forest, Qld, P.I. Forster 25088, BRI AQ0678653 |
| *Phebalium* *woombye* (Bailey) Domin | AF066822 | AF066852 | Chase *et al.* (1999) |
| *Phellodendron* *amurense* Rupr. | AF066804 | AF066833 | Chase *et al.* (1999) |
| *Philotheca* *brevifolia* (Endl.) Paul G.Wilson | AF156882[[4]](#footnote-5) | AF156883Error: Reference source not found | Chase *et al.* (1999) |
| *Philotheca* *buxifolia* (Sm.) Paul G.Wilson subsp. *buxifolia* | JN987130 | JN987048 | Cultivated Rosanna Vic., M.J. Bayly 1959, MELU 105853 |
| *Philotheca* *deserti* (E.Pritz.) Paul G.Wilson subsp. *deserti* | JN987131 | JN987049 | Paynes Find, WA, M.J. Bayly 1919, MEL |
| *Philotheca* *fitzgeraldii* (C.R.P.Andrews) Paul G.Wilson | JN987129 | JN987047 | Near intersection of Lake King-Norseman Rd & Coolgardie-Esperance Hwy, WA, M.J. Bayly 1942, MEL |
| *Philotheca* *spicata* (A.Rich.) Paul G.Wilson | JN987132 | JN987050 | Alexander Morrison National Park, WA, M.J. Bayly 1907, MEL |
| *Picrella glandulosa* T.G.Hartley **1** | JN987133 | JN987051 | Aoupinié, New Caledonia, M.J. Bayly 2106, MEL, BRI, NOU |
| *Picrella glandulosa* T.G.Hartley **2** | JN987134 | JN987052 | Farino, New Caledonia, J. Munzinger 2078, NOU |
| *Picrella ignambiensis* (Guillaumin) T.G.Hartley & Mabb. | JN987135 | JN987053 | Aoupinié, New Caledonia, M.J. Bayly 2113, MEL, BRI, NOU |
| *Picrella trifoliata* Baill. | JN987136 | JN987054 | Ouaménie, New Caledonia, J. Munzinger 3494, NOU |
| *Pilocarpus pennatifolius* Lem. | AF066809 | AF066825 | Chase *et al.* (1999) |
| *Pitaviaster* *haplophyllus* (F.Muell.) T.G.Hartley | JN987137 | JN987055 | CSIRO arboretum, Atherton, ex Swiper’s Logging Area, Qld, M.J. Bayly 1885, MELU 105869 |
| *Pleiospermium* *alatum* Wight & Arn. | AF066821 | AF066850 | Chase *et al.* (1999) |
| *Ruta graveolens* L. | RGU39281 | AF035913 | Gadek et al. 1996, Savolainen et al. |
| *Sarcomelicope argyrophylla* Guillaumin | JN987138 | JN987056 | Haute vallée de la Ni, Kouakoué, New Caledonia, J. Munzinger 2019, NOU |
| *Sarcomelicope* *simplicifolia* (Endl.) subsp. *neo-scotica* (P.S.Green) T.G.Hartley **3** | JN987140 | JN987058 | Pindai, New Caledonia, M.J. Bayly 2087, MEL, BRI, NOU |
| *Sarcomelicope* *simplicifolia* (Endl.) T. G. Hartley **1** | AF066817 | AF066845 | Chase *et al.* (1999) |
| *Sarcomelicope* *simplicifolia* (Endl.) T.G.Hartley subsp. *simplicifolia* **2** | JN987139 | JN987057 | Transit Hill, Lord Howe Island, MELU 105912 |
| *Skimmia* *anquetilia* N.P. Taylor & H.K. Airy Shaw | AF066818 | AF066846 | Chase *et al.* (1999) |
| *Zanthoxylum* *brachyacanthum* F.Muell. | JN987141 | JN987059 | Jack Smith's Scrub Conservation Park, Qld, P.I. Forster 33073, BRI |
| *Zanthoxylum monophyllum* (Lam.) P. Wilson | ZMU39282 | AF035919 | Gadek et al. 1996, Savolainen et al. |
| *Zanthoxylum* *pinnatum* (J.R.Forst. & G.Forst.) W.R.B.Oliv. | JN987142 | JN987060 | Transit Hill, Lord Howe Island, P.Y. Ladiges, MELU 105913 |
| *Zanthoxylum* sp. (J.W.Clayton 15) | EU042976 | EU042768 | Clayton *et al.* (2007) |
| *Zanthoxylum* sp. (M. W. Chase 1348, K) | AF066815 | AF066843 | Chase *et al.* (1999) |
| *Zieria* *arborescens* Sims subsp. *arborescens* | JN987143 | JN987061 | Starlings Gap, Vic., M.J. Bayly 1868, MELU |
| *Zieria chevalieri* Virot | JN987144 | JN987062 | Mt Kaala, New Caledonia, M.J. Bayly 2119, MEL, BRI, NOU |
| *Zieria* *madida* Duretto & P.I.Forst. | JN987145 | JN987063 | Thornton Peak, Qld, A. Ford 5320, BRI |

Abbreviations: BRI, Queensland Herbarium, Brisbane; MEL, National Herbarium of Victoria, Melbourne. MELU, University of Melbourne Herbarium, Melbourne; NOU IRD herbarium in Nouméa; NSW, New South Wales, Australia; NT, Northern Territory, Australia; Qld, Queensland, Australia; Vic., Victoria, Australia; WA, Western Australia.

## References

1. Chase MW, Morton CM, Kallunki JA (1999) Phylogenetic relationships of Rutaceae: a cladistic analysis of the subfamilies using evidence from RBC and ATP sequence variation. Am J Bot 86: 1191–1199.

2. Bausher M, Singh N, Lee S-B, Jansen R, Daniell H (2006) The complete chloroplast genome sequence of *Citrus sinensis* (L.) Osbeck var 'Ridge Pineapple': organization and phylogenetic relationships to other angiosperms. BMC Pl Biol 6: 21.

3. Clayton J, Fernando E, Soltis P, Soltis DE (2007) Molecular phylogeny of the Tree-of-Heaven family (Simaroubaceae) based on chloroplast and nuclear markers. Int J Pl Sci 168: 1325–1339.

1. As *Severinia buxifolia* [↑](#footnote-ref-2)
2. As *Eremocitrus glauca* [↑](#footnote-ref-3)
3. As *Fortunella japonica* [↑](#footnote-ref-4)
4. As *Eriostemon brevifolius*. This species is fairly uncommon and the name *E. brevifolius* has sometimes been applied to other small-leaved members of *Philotheca* sect. *Philotheca* from eastern Australia, e.g., *P. angustifolia* and *P. difformis*. The voucher cited by Chase et al. , with no details of wild provenance, could not be found, either physically or in the electronic database of the CBG herbarium (now part of CANB), so we have not been able to verify the identity of the species. [↑](#footnote-ref-5)
