# Supplementary material for: Major Clades of Australasian Rutoideae (Rutaceae) Based on rbcL and atpB Sequences
Source: PLoS One. 2013 Aug 13;8(8):e72493. doi: 10.1371/journal.pone.0072493 (PMC3742607; doi:10.1371/journal.pone.0072493)
Supplement: Table S2 — Details of additional sequences (GenBank numbers) included in molecular dating analyses. (DOCX) [file pone.0072493.s004.docx]

## Table S2 Details of additional sequences (GenBank numbers) included in molecular dating analyses.

Numbers not preceded by a letter prefix are GenBank ‘GI’ numbers.

| **Taxa** | **rbcL** | **atpB** |
| --- | --- | --- |
| **Sapindales** |  |  |
| **Rutaceae** |  |  |
| *Bergera koenigii* | AB505905 | EF118832 |
| *Bottegoa insignis* | AJ402931 | FR747871 |
| *Cedrelopsis gracilis* | FR747839 | FR747873 |
| *Cneorum pulverulentum* | FR747836 | AF209567 |
| *Dictyoloma vandellianum* | AF066823 | AF066853 |
| *Harrisonia abyssinica* | FR747832 | FR747868 |
| *Harrisonia brownii* | FR747828 | FR74786 |
| *Micromelum minutum* | AB505902 | EF118889 |
| *Murraya paniculata* | U38860 | EF118891 |
| *Orixa japonica* | HE588085 | HE588080 |
| *Ptelea baldwinii* | HE588086 | HE588081 |
| *Spathelia brittonii* | FR747847 | FR747881 |
| *Spathelia excelsa* | AF066798 | AF066854 |
| *Tetradium meliifolium* | FN599466 | FN599858 |
| *Tetradium ruticarpum* | GQ436747 | – |
| *Toddalia asiatica* | JQ933504 | EF118900 |
| *Triphasia trifolia* | AB505911 | EF118902 |
| **Simaraoubaceae** |  |  |
| *Ailanthus altissima* | AY128247 | AF035895 |
| *Amaroria soulameoides* | U38923 | AF066856 |
| *Brucea javanica* | EU042986 | EU042778 |
| *Castela erecta* | EU042990 | EU042781 |
| *Eurycoma apiculata* | EU042995 | EU042786 |
| *Hannoa chlorantha* | EU042990 | EU042781 |
| *Holacantha emoryi* | EU043002 | EU042793 |
| *Leitneria floridana* | AF062003 | EU042794 |
| *Nothospondias staudtii* | EU043004 | EU042795 |
| *Odyendyea gabonensis* | EU043005 | EU042796 |
| *Perriera madagascariensis* | EU043007 | EU042798 |
| *Picrasma javanica* | EU043011 | EU042802 |
| *Picrolemma sprucei* | EU043014 | EU042804 |
| *Quassia amara* | EU043017 | EU042807 |
| *Samadera indica* | EU043020 | EU042810 |
| *Simaba guianensis* | EU043034 | EU042824 |
| *Simarouba berteroana* | EU546231 | EU546249 |
| *Simarouba glauca* | AY128252 | AJ235602 |
| *Soulamea* sp. Robertson 2529 | EU043042 | EU042832 |
| **Meliaceae** |  |  |
| *Melia azedarach* | EU042973 | EU042764 |
| *Nymania capensis* | AY128238 | AF066855 |
| *Swietenia macrophylla* | AY128241 | AF066857 |
| *Trichilia emetica* | TEU39082 | AF066851 |
| **Nitrariaceae** |  |  |
| *Nitraria* | 88174765 | - |
| **Anacardiaceae** |  |  |
| *Schinus molle* | U39270 | AF035914 |
| **Burseraceae** |  |  |
| *Bursera microphylla* | GU246027 | GU246055 |
| **Other eudicot orders** |  |  |
| *Angophora floribunda* | KC180804 | KC180804 |
| *Arabidopsis* | NC_000932 | NC_000932 |
| *Austromuellera* | DQ875865 | AF060414 |
| *Bellendena montana* | DQ875821 | AF060390 |
| *Berberidopsis* | EU002274 | HQ843255 |
| *Buxus* | 81230666 | 5001578 |
| *Campanula* | AY655147 | EU437664 |
| *Cucurbita* | 17135916 | 14718023 |
| *Dillenia* | L01903 | AY788268 |
| *Drosera* | L01909 | AY096110 |
| *Embothrium coccineum* | DQ875857 | AF060429 |
| *Eucalyptus erythrocorys* | KC180799 | KC180799 |
| *Eucalyptus saligna* | KC180790 | KC180790 |
| *Euptelea* | 21654915 | 33327748 |
| *Fagus grandifolia* | HQ590099 | AY147105 |
| *Geranium* | 93133009 | 197132377 |
| *Gossypium* | 11562 | 4995178 |
| *Gunnera* | 11323502 | 6017788 |
| *Heisteria* | 112408851 | 8452661 |
| *Helianthus* | 4249678 | 6687661 |
| *Heteropyxis* | U26326 | AF209597 |
| *Hypericum perforatum* | JX664053 | JX663806 |
| *Ilex* | EF590536 | AF471679 |
| *Itea* | JN102266 | AF093383 |
| *Ixerba* | 4530131 | 14718088 |
| *Juglans nigra* | AF206785 | AF209609 |
| *Lotus* | U74213 | AP002983 |
| *Malpighia* | 14599571 | 8439434 |
| *Meliosma* | 17135983 | 14718125 |
| *Myrothamnus flabellifolius* | AF060707 | AF093386 |
| *Myrtus communis* | AF294254 | JF268426 |
| *Nothofagus* | 50604300 | 37729419 |
| *Opisthiolepis heterophylla* | DQ875860 | AF060432 |
| *Opuntia* | AY875233 | HQ843258 |
| *Paeonia* | 9909878 | 8452716 |
| *Petrophile* | 4098553 | 3850921 |
| *Pittosporum* | L11202 | AF528857 |
| *Platanus occidentalis* | DQ875820 | AF528858 |
| *Polygala* | 148590247 | 8517655 |
| *Polygonum* | DQ006118 | AJ235569 |
| *Psiloxylon mauritianum* | U26333 | – |
| *Ranunculus* | 255995478 | 89242535 |
| *Ribes aureum* | L11204 | AF528859 |
| *Ricinus* | AY788188 | AY788253 |
| *Stellaria* | AF206823 | AF209680 |
| *Thymelaea hirsuta* | Y15151 | AJ235626 |
| *Toronia* | DQ875823 | AF060392 |
| *Trochodendron* | 7240475 | 157689169 |
| *Viola* | DQ006129 | 257852979 |
| *Vitis* | L01960 | DQ424856 |
| *Wittsteinia* | X87399 | AJ318986 |
