# Supplementary material for: Major Clades of Australasian Rutoideae (Rutaceae) Based on rbcL and atpB Sequences
Source: PLoS One. 2013 Aug 13;8(8):e72493. doi: 10.1371/journal.pone.0072493 (PMC3742607; doi:10.1371/journal.pone.0072493)
Supplement: Table S3 — Fossil calibrations used in molecular dating analyses. (DOCX) [file pone.0072493.s005.docx]

## Table S3 Fossil calibrations used in molecular dating analyses.

MRCA= most recent common ancestor. Age estimates include means plus upper and lower bounds of 95% highest posterior density intervals

| **Calibration No.** | **Taxonomic group** | **Fossil** | **References** |  | **MRCA constraint** |  | **Minimum age constraint** | **Estimated age from molecular dating analysis** |
| --- | --- | --- | --- | --- | --- | --- | --- | --- |
|  | **Rutaceae** |  |  |  |  |  |  |  |
| 1 | *Skimmia* (Rutaceae) | *Skimmia tortonica* | [[1](#_ENREF_1),[2](#_ENREF_2),[3](#_ENREF_3)] |  | *Skimmia*/*Dictamnus* (crown) |  | 7.4 | (8) 17 (26.6) |
| 2 | *Tetradium*/*Phellodendron* (Rutaceae) | *Euodia costata* | [[1](#_ENREF_1),[4](#_ENREF_4),[5](#_ENREF_5)] |  | *Tetradium*/*Phellodendron* (stem)* |  | 55.5 | (55.5) 57.2 (60.3) |
| 3 | *Clausena* (Rutaceae) | *Clausena* | [[1](#_ENREF_1),[6](#_ENREF_6)] |  | *Clausena*/*Bergera* (crown) |  | 27.25 | (27.25) 31.5 (38) |
|  | **Simaraubaceae** |  |  |  |  |  |  |  |
| 4 | *Ailanthus* (Simaraubaceae) | *Ailanthus* | [[1](#_ENREF_1),[7](#_ENREF_7),[8](#_ENREF_8)] |  | *Ailanthus*/*Simaba* (crown) |  | 52 | (52) 53.42 (56.2) |
|  | **Other Eudicots** |  |  |  |  |  |  |  |
| 5 | Malvales *s.l.* | *Parbombacaceoxylon* | [[9](#_ENREF_9),[10](#_ENREF_10)] |  | *Gossypium*/*Thymelaea* (crown) |  | 65.5 | (65.5) 75.1 (87.5) |
| 6 | Myrtales | *Myrtaceidites* (=*Syncolporites*) | [[11](#_ENREF_11),[12](#_ENREF_12),[13](#_ENREF_13)] |  | *Angophora*/*Eucalyptus*/*Myrtus*/*Psiloxylon*/*Heteropyxis* (crown) |  | 86 | (86) 88.5 (92.9) |
| 7 | *Eucalyptus* (Myrtaceae) | *Eucalyptus* | [[14](#_ENREF_14)] |  | *Eucalyptus erythrocorys*/*E. saligna* (crown) |  | 51.9 | (51.9) 54.8 (60.4) |
| 8 | Hypericaceae/Clusiaceae (Clusiaceae) | *Paleoclusia* | [[9](#_ENREF_9),[15](#_ENREF_15)] |  | *Hypericum*/*Malphigia*  (crown) |  | 89 | (89) 92.4 (97.7) |
| 9 | Core Fagales | *Protofagacea allonensis* | [[16](#_ENREF_16),[17](#_ENREF_17)] |  | *Fagus*/*Juglans* (crown) |  | 83.5 | (83.5) 85.2 (88.4) |
| 10 | Iteaceae | unnamed | [[9](#_ENREF_9),[18](#_ENREF_18)] |  | *Itea*/*Ribes* (crown) |  | 89.3 | (89.3) 95.9 (104.5) |
| 11 | Gunneraceae | *Retitricolpites microreticulatus* | [[9](#_ENREF_9),[12](#_ENREF_12)] |  | *Gunnera*/*Myrothamnus* (crown) |  | 88.2 | (88.2) 96.8 (107.7) |
| 12 | Buxaceae | unnamed | [[19](#_ENREF_19)] |  | *Buxus*/*Trochodendron* (crown) |  | 94 | (95.7) 110 (121.6) |
| 13 | Platanaceae | *Plantanocarpus* *brookensis*, *Aquia brookensis* | [[20](#_ENREF_20),[21](#_ENREF_21),[22](#_ENREF_22)] |  | *Platanus*/Proteaceae (crown) |  | 108 | (108) 111.9 (116.7) |
| 14 | Proteaceae | *Triorites africaensis* | [[20](#_ENREF_20),[23](#_ENREF_23),[24](#_ENREF_24)] |  | *Toronia*/*Bellendena*/*Austromuellera*/*Petrophile*/*Embothrium*/ *Opisthiolepis* (crown) |  | 93 | (93) 96.3 (101.75) |
| 15 | Stem Embothriinae (Proteaceae) | *Propylipollis ambiguus* | [[20](#_ENREF_20),[23](#_ENREF_23),[25](#_ENREF_25),[26](#_ENREF_26)] |  | *Embothrium*/*Opisthiolepis* (crown) |  | 70 | (70) 73.6 (79.8) |

* Two placements for this fossil have been discussed in the literature [[1](#_ENREF_1)]. The scheme used here is the more conservative of the two, but trials (not shown) assigning this fossil to the *Tetradium*/*Phellodendron* crown had little influence on age estimates for nodes of interest in this study.

## References

1. Appelhans MS, Keßler PJA, Smets E, Razafimandimbison SG, Janssens SB (2012) Age and historical biogeography of the pantropically distributed Spathelioideae (Rutaceae, Sapindales). J Biogeogr 39: 1235-1250.

2. Palamarev D, Usunova K (1970) Morphologisch-anatomischer Nachweis der Gattung Skimmia in der Tertiarflora Bulgariens. Dokladi na Bulgarskata Akademiya Naukite 23: 835–838.

3. Salvo G, Ho S, Rosenbaum G, Ree R, Conti E (2010) Tracing the temporal and spatial origins of island endemics in the mediterranean region: a case study from the citrus family (*Ruta* L., Rutaceae). Syst Biol 59: 705–722.

4. Hartley T (2000) On the taxonomy and biogeography of *Euodia* and *Melicope* (Rutaceae). Allertonia 8: 1–328.

5. Tiffney B (1981) *Euodia costata* (Chandler) Tiffney, (Rutaceae) from the Eocene of southern England. Paläont Z 55: 185-190.

6. Pan AD (2010) Rutaceae leaf fossils from the Late Oligocene (27.23 Ma) Guang River flora of northwestern Ethiopia. Review of Palaeobotany and Palynology 159: 188-194.

7. Corbett SL, Manchester SR (2004) Phytogeography and fossil history of *Ailanthus* (Simaroubaceae). Int J Pl Sci 165: 671-690.

8. Pfeil BE, Crisp MD (2008) The age and biogeography of *Citrus* and the orange subfamily (Rutaceae: Aurantioideae) in Australasia and New Caledonia. Am J Bot 95: 1621–1631.

9. Bell CD, Soltis DE, Soltis PS (2010) The age and diversification of the angiosperms re-revisited. Am J Bot 97: 1296-1303.

10. Wheeler EF, Lee M, Matten LC (1987) Dicotyledonous woods from the Upper Cretaceous of southern Illinois. Bot J Linn Soc 95: 77-100.

11. Herngreen GFW (1975) An Upper Senonian pollen assemblage of borehole 3-PIA-10-AL state of Alagoas, Brazil. Pollen Spores 17: 93-140.

12. Muller J (1981) Fossil pollen records of extant angiosperms. Botanical Review 47: 1-142.

13. Sytsma KJ, Litt A, Zjhra ML, Pires JC, Nepokroeff M, et al. (2004) Clades, clocks, and continents: historical and biogeographical analysis of Myrtaceae, Vochysiaceae, and relatives in the southern hemisphere. Int J Pl Sci 165: S85-S105.

14. Gandolfo MA, Hermsen EJ, Zamaloa MC, Nixon KC, González CC, et al. (2011) Oldest known *Eucalyptus* macrofossils are from South America. PLoS ONE 6: e21084.

15. Crepet W, Nixon K (1998) Fossil Clusiaceae from the late Cretaceous (Turonian) of New Jersey and implications regarding the history of bee pollination. Am J Bot 85: 1122.

16. Herendeen PS, Crane PR, Drinnan AN (1995) Fagaceous flowers, fruits, and cupules from the Campanian (Late Cretaceous) of central Georgia, USA. Int J Pl Sci 156: 93-116.

17. Sauquet H, Ho SYW, Gandolfo MA, Jordan GJ, Wilf P, et al. (2012) Testing the Impact of Calibration on Molecular Divergence Times Using a Fossil-rich Group: The Case of *Nothofagus* (Fagales). Syst Biol 61: 289-313.

18. Hermsen EJ, Gandolfo MA, Nixon KC, Crepet WL (2003) *Divisestylus gen*. *nov*. (*aff*. Iteaceae), a fossil saxifrage from the Late Cretaceous of New Jersey, USA. Am J Bot 90: 1373-1388.

19. Drinnan AN, Crane PR, Friis EM, Pedersen KR (1991) Angiosperm flowers and tricolpate pollen of Buxaceous affinity from the Potomac Group (Mid-Cretaceous) of eastern North America. Am J Bot 78: 153-176.

20. Barker NP, Weston PH, Rutschmann F, Sauquet H (2007) Molecular dating of the ‘Gondwanan’ plant family Proteaceae is only partially congruent with the timing of the break-up of Gondwana. J Biogeogr 34: 2012-2027.

21. Crane PR, Pedersen KR, Friis EM, Drinnan AN (1993) Early Cretaceous (Early to Middle Albian) platanoid inflorescences associated with sapindopsis leaves from the Potomac Group of eastern North America. Syst Bot 18: 328-344.

22. Drinnan A, Crane P, Hoot S (1994) Patterns of floral evolution in the early diversification of non-magnoliid dicotyledons (eudicots). In: Endress P, Friis E, editors. Early Evolution of Flowers: Springer Vienna. pp. 93-122.

23. Dettmann ME, Jarzen DM (1998) The Early History of the Proteaceae in Australia: the Pollen Record. Aust Syst Bot 11: 401-438.

24. Ward JV, Doyle JA (1994) Ultrastructure and relationships of mid-Cretaceous polyforate and triporate pollen from northern Gondwana. In: Kurmann MH, Doyle JA, editors. Ultrastructure of fossil spores and pollen: Royal Botanic Gardens, Kew. pp. 161-172.

25. Feuer S (1990) Pollen morphology of the Embothrieae (Proteaceae) II. Embothriinae (*Embothrium*, *Oreocallis*, *Telopea*). Grana 29: 19-36.

26. Weston P, Crisp M (1994) Cladistic biogeography of waratahs (Proteaceae, Embothrieae) and their allies across the pacific. Aust Syst Bot 7: 225-249.
